# Chromosome-level genome assembly of Tarim red deer, *Cervus elaphus yarkandensis*

**DOI:** 10.1038/s41597-020-0537-0

**Published:** 2020-06-19

**Authors:** Hengxing Ba, Zexi Cai, Haoyang Gao, Tao Qin, Wenyuan Liu, Liuwei Xie, Yaolei Zhang, Binyu Jing, Datao Wang, Chunyi Li

**Affiliations:** 1 Institute of Special Wild Economic Animals and Plants, Chinese Academy of Agricultural Sciences, Changchun, 130112 China; 2 Changchun Sci-Tech University, Changchun, 130600 China; 3 Center for Quantitative Genetics and Genomics, Department of Molecular Biology and Genetics, Aarhus University, 8830 Tjele, Denmark; 4 BGI-Qingdao, BGI-Shenzhen, Qingdao, 266032 Shandong Province China; 5 Xinjiang Company Ltd of Houshi Biological Science and Technology, 830002, Urumchi, China

**Keywords:** Genome informatics, Genome, DNA sequencing, Zoology

## Abstract

Tarim red deer (*Cervus elaphus yarkandensis*) is the only subspecies of red deer (of 22 subspecies) from Central Asia. This species is a desert dweller of the Tarim Basin of southern Xinjiang, China, and exhibits some unique adaptations to the dry and extreme hot climate. We report here the assembly of a Tarim red deer genome employing a 10X Genomics library, termed CEY_v1. Our genome consisted of 2.6 Gb with contig N50 and scaffold N50 of 275.5 Kb and 31.7 Mb, respectively. Around 96% of the assembled sequences were anchored onto 34 chromosomes based on the published high-quality red deer genetic linkage map. More than 94% BUSCOs complete genes (including 90.5% single and 3.6% duplicated ones) were detected in the CEY_v1 and 20,653 genes were annotated. The CEY_v1 is expected to contribute to comparative analysis of genome biology, to evolutionary studies within Cervidae, and to facilitating investigation of mechanisms underlying adaptation of this species to the extreme dry and hot climate.

## Abstract (summary)

**Table:** 

Measurement(s)	DNA • genome • sequence_assembly • sequence feature annotation
Technology Type(s)	DNA sequencing • sequence assembly process • sequence annotation
Sample Characteristic - Organism	Cervus elaphus

Machine-accessible metadata file describing the reported data: 10.6084/m9.figshare.12410564

## Background & Summary

Cervidae is the second largest family in Ruminantia (second to Bovidae) and consists of 56 species^1^. Along with the common distinct attributes of ruminants (i.e. even-toe, multi-chambered stomach and headgear), males in Cervidae grow deciduous antlers (except for antlerless Chinese water deer and antlers in both sexes in reindeer)^2^. Deer are excellent models for studying evolution, biodiversity, interspecies hybridization^3,4^, social organization (i.e. hierarchical status)^5^, unique organ development (i.e. fully regenerable antlers)^6^ and habitat selection (extreme cold vs extreme hot)^7,8^.

**Fig. 1:**
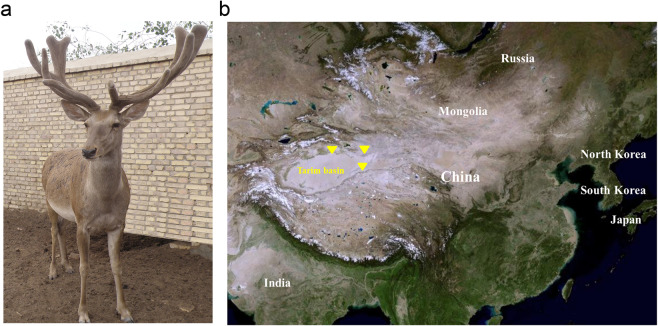
Photograph and location of the Tarim red deer selected in this study. (**a**) A photograph of an adult male Tarim red deer individual, from which blood samples were collected for genome sequencing. (**b**) A natural distribution map of Tarim red deer (yellow arrowhead).

Red deer (*Cervus elaphus*) is the most studied species in Cervidae and consists of 22 extant subspecies^9^. Of these subspecies, eight are found in China, and three of these Chinese subspecies inhabit Xinjiang in northwest China: Tianshan red deer (*C. e. songaricus* Severzov, 1872), Altai red deer (*C. e. sibiricus* Severzov, 1873) and Tarim red deer (*C. e. yarkandensis* Blanford, 1892)^10,11^. Tarim red deer (Fig. 1a) is the only subspecies of red deer resident in Central Asia, a proposed site of origin for the genus *Cervus*^12^. This deer subspecies tolerates the extreme dry (mean annual evaporation is 45.8 times more than the precipitation, and mean rainfall is 18.6 mm/year) and hot (average temperature in summer is 32.7 °C) desert environment of the Tarim Basin of southern Xinjiang (Fig. 1b), China^10^. Although little is known about the biology of this deer subspecies, it is likely to have evolved mechanisms to adapt to this hostile habitat. Recently, Tarim red deer has been classified as an endangered species by IUCN and has been included in the China Red Data Book of Endangered Animals, as the population in its native habitat has been declining^10^.

Whole genome sequencing has become an increasingly popular technology to explore taxonomy, evolution, biological phenomena and distinct attributes of organisms at a genomic level, as opposed to morphological, histological and other means^13,14^. Chen *et al*.^15^recently published a paper in the prestigious journal “Science”, within which 44 ruminant genomes were sequenced, including 6 deer species^15^. To date, 13 draft deer genomes have been reported, covering four deer subfamilies: Cervinae (4)^15–19^, Muntiacinae (3)^15,20^, Hydropotinae (1)^15^, and Odocoileinae (5)^21–26^. However, genomes of the most deer species (43) remain yet to be sequenced, including some of the more important deer species with economic value, such as sika deer and red deer (production of precious Chinese medicines, velvet antler). Consequently, the evolution of the distinctive features of these deer species has not been resolved at the genetic level, for example, the adaptation of Tarim red deer to its extremely dry and hot environment. In addition, the quality of these published deer genomes is still not comparable to some other ruminants, such as bovine^14^. Therefore, whether these deer genomes can be served as a reference genome for relevant future studies is questionable.

This paper reports a high quality Tarim red deer genome, which was generated through the combination of sequences created in the present study using the 10X Genomics GemCode platform with the previously published genetic linkage map data^27,28^; and is termed here CEY_v1. The final CEY_v1 was 2.60 Gb and consisted of 19,010 scaffolds (scaffolds > = 1 Kb) with 2.21% missing bases, with the contig N50 and scaffold N50 of 275.5 Kb and 31.7 Mb respectively. A total of 269 scaffolds, accounting for 96% of CEY_v1, were anchored onto 34 chromosomes. Almost 100% of the predicted genes (20,652) were annotated using biological databases. We believe that this high-quality reference genome of CEY_v1 will provide a valuable resource for future studies to Tarim red deer in particular, and to Cervidae and even Ruminantia in general, as well as to shed light on the molecular mechanism of animal adaptation to extreme hostile environments.

## Methods

### Ethics statement

Blood sampling carried out in this study was approved by the Animal Ethics Committee of Institute of Special Wild Economic Animals and Plants, Chinese Academy of Agricultural Sciences (CAAS2017-06).

### Genomic DNA extraction

A 4-year-old semi-domesticated male Tarim red deer (Fig. 1a) from the Korla region (Xinjiang Autonmous Region, China) was selected for blood sampling (via jugular using EDTA vacuum tubes). The blood sample was stored at −80 °C until DNA extraction. Genomic DNA was extracted and purified using QIAamp Blood DNA midi kit (Qiagen, Valencia, CA, USA).

### Construction of 10x Genomics library

The Genomic DNA concentrations were measured using a Qubit® 2.0 Fluorometer (Life Technologies). Their quality was assessed using 1% gel electrophoresis to determine suitability for 10x Chromium library construction (10x Genomics, San Francisco, USA). Genomic DNA (total of 1.2 ng) was used for library construction after passing quality assessment according to the manufacturer’s instructions without size-selection. The barcode sequencing libraries were quantified using qPCR (KAPA Biosystems Library Quantification Kit for Illumina platforms). Finally, sequencing was conducted with 2 × 150 paired-end reads in two lanes using the Illumina HiSeq. 4000 platform at BGI (China).

### Genome sequencing and *de novo* assembly

In total, 195 Gb sequencing data were generated from the Illumina paired-end sequencing. After low-quality reads were removed using NGS QC Toolkit^29^ with default parameters, 183.5 Gb of clean bases were obtained for *de novo* assembly using the Supernova (v2.0.1, 10x Genomics) assembler. The estimated genome size was 2.86 Gb with 63-fold raw and 43-fold effective coverage. The final size of our assembled draft genome was 2.60 Gb, with 19,010 scaffolds (scaffolds >  = 1 Kb) with 2.21% missing bases, with contig N50 and scaffold N50 of 275.5 Kb and 31.7 Mb respectively.

### Anchorage of the genome assembly onto chromosomes

**Fig. 2:**
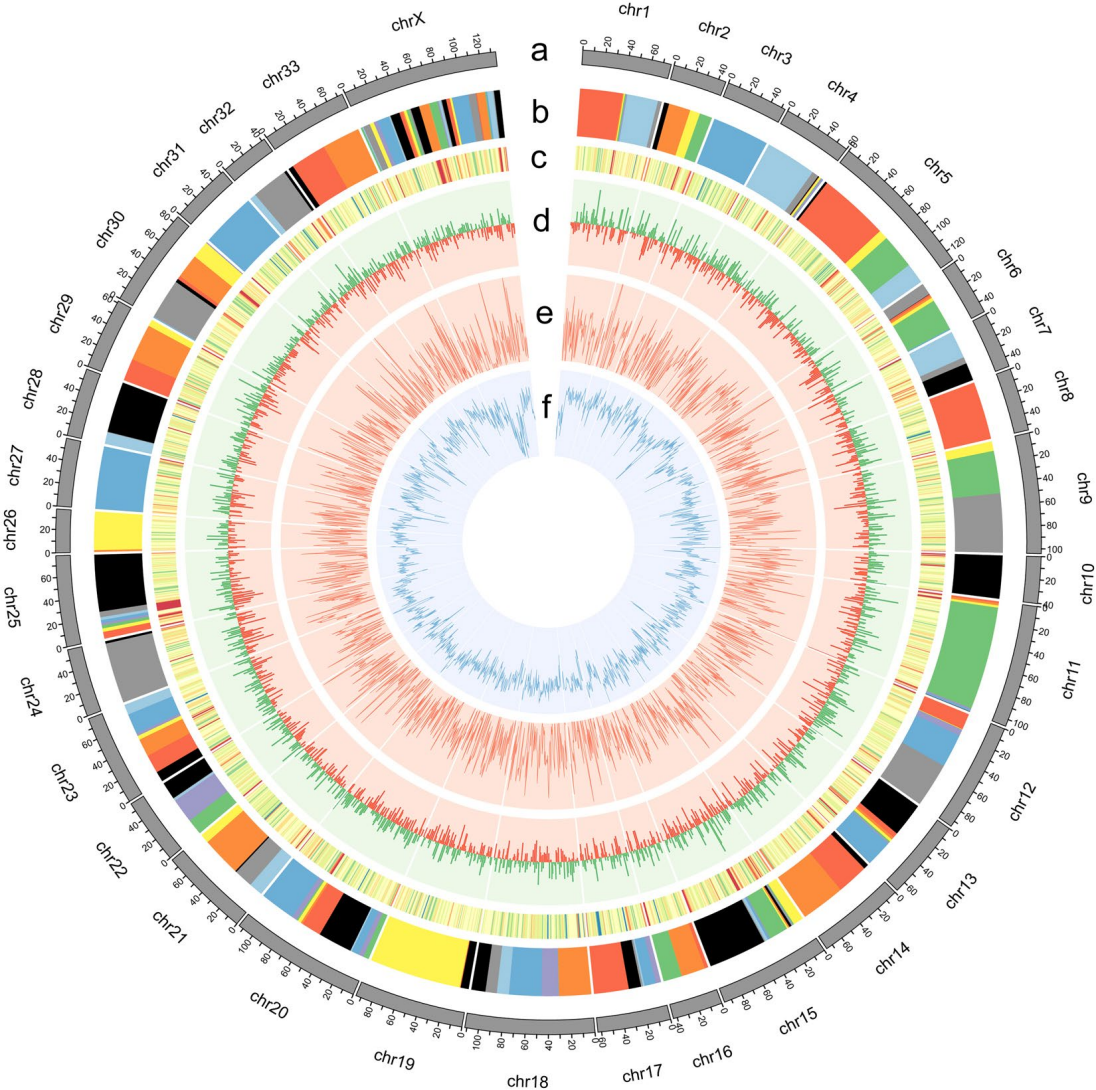
Circos plot showing 34 chromosomes of CEY_v1. (**a**) chromosome length in Mb unit; (**b**) arrangement of the scaffolds (>1 Mb) in random colors within each chromosome; (**c**) the heatmap mapped SNPs number within 1 Mb window, ranging from 0 to 60; (**d**) histogram showing the GC skewer of 1 Mb windows with 1 Kb step size; (**e**) line plot of gene density for 1 Mb windows, and (**f**) line plot of repeat density for 1 Mb windows.

We further anchored these scaffolds onto chromosomes using ALLMAPS (v0.8.4)^30^ based on the published high-quality red deer genetic linkage map^27,28^. This published map consists of 34 sex-averaged linkage groups including a total of 38,083 SNP markers based on the haploid chromosome number for red deer with 2,740 cM in combined length. The locations of SNPs were obtained by mapping the probe sequences (150 bp on both ends) of these SNP markers to our assembled sequences using BWA (v0.7.17)^31^. The probes with multiple alignments were removed. At the end, we successfully placed 38,042 (99.89%) uniquely-mapped SNPs onto 34 chromosomes (Fig. 2). The information of the location of the SNPs in our assembly were retained for downstream analysis. To take advantage of the public availability of female and male genetic maps, the two maps were assigned equal weight and merged. Overall, we anchored 269 scaffolds onto 34 chromosomes, representing 95.9% of the total assembled genome. Of these scaffolds, 160 had more than two markers and were oriented, representing 94.2% of CEY_v1 (Fig. 2 and Table 1). In CEY_v1, three small autosomes (i.e. chr 3, 8 and 31) contained only one large scaffold, whereas sex chromosome X had the highest number of scaffolds (Fig. 2). Given that the genetic linkage map is from a closely-related subspecies, we arbitrarily set 100 bp for the size of gaps that were unknown.

**Table 1:** Statistics of chromosome anchoring based on the SNP markers.

	Anchored	Oriented	Unplaced
Markers (unique)	38,083	37,606	106
Markers per Mb	15.5	15.5	1
N50 Scaffolds	28	28	0
Scaffolds	269	160	18,740
Scaffolds with 1 marker	63	0	91
Scaffolds with 2 markers	14	2	4
Scaffolds with 3 markers	9	2	1
Scaffolds with > = 4 markers	183	156	1
Total bases	2,490,596,933 (95.90%)	2,441,137,212 (94.2%)	106,169,671 (4.10%)

### Identifying Y chromosome scaffolds

Because of its repetitive nature, assembling the Y chromosome is particularly challenging. Using a previous Y chromosome assemblies from cattle^14^ and red deer^19^, we detected 37 scaffolds that are likely to be located on the Y chromosome using BLAST tools (E-value ≤ 1e^−50^). These encompass a total length of 5.15 Mb. Among the 33 genes structurally annotated on those scaffolds, four were identified as SRY, TSPY1, TSPY3 and ZFY. In humans, these four genes are linked to the Y chromosome, confirming the location of the four Tarim red deer scaffolds identified on the Y chromosome.

### Annotation of repeat sequences

We annotated the repeat sequences in CEY_v1 using both *de novo* predictions and homology-based searching in the known repeat database. RepeatModeler (v1.0.11)^32^ and LTR_FINDER (v1.0.5)^33^ were used to construct the *de novo* repeat library. We used RepeatMasker (v3.3.0, http://www.repeatmasker.org/) with the RepBase (v17.01, http://www.girinst.org/repbase)^34^ transposable element (TE) library to identify known repeats in our genome. In addition, RepeatProteinMask in RepeatMasker (v3.3.0) was used to identify the TE proteins. Tandem Repeats Finder (TRF, v4.07)^35^ was used to identify the tandem repeats. The results showed that CEY_v1 contained a total of 1.09 Gb of non-redundant repetitive sequences, which accounted for 42.4% of the whole genome (Fig. 2 and Table 2). The main elements were LINEs, which accounted for 37.8% (980 Mb) of the whole genome (Table 3).

**Table 2:** Prediction of repeat elements in the Tarim red deer genome.

Type	Repeat Size(bp)	% of genome
TRF	26,065,074	1.00
RepeatMasker	836,426,458	32.21
RepeatProteinMask	431,640,750	16.62
*De novo*	988,599,789	38.07
Total	1,099,992,590	42.36

**Table 3:** Statistics of repeat elements in the Tarim red deer genome.

	*De novo*		Repbase TEs		TE Proteins		Combined TEs	
	Length (bp)	% in Genome	Length (bp)	% in Genome	Length (bp)	% in Genome	Length (bp)	% in Genome
DNA	765,397	0.03	26,322,675	1.01	655,292	0.25	26,729,330	1.03
LINE	855,277,270	32.94	640,898,202	24.68	423,761,737	16.32	980,437,996	37.76
SINE	281,327	0.01	109,276,352	4.21	0	0.00	109,493,480	4.22
LTR	247,139,539	9.52	73,669,154	2.84	7,252,671	0.28	303,709,517	11.70
Other	0	0.00	192	0.00	444	0.00	636	0.00
Unknown	3,083,692	0.12	0	0.00	0	0.00	3,083,692	0.12
Total	988,599,789	38.07	836,426,458	32.21	431,640,750	16.62	1,086,749,836	41.85

### Gene prediction and functional annotation

After the repeat sequences were masked, *de novo* prediction was carried out with the *Bos taurus* training set based on default parameters using Augustus (v3.2.1)^36^. For homology prediction, protein sequences from six mammals (*Bos taurus*, *Homo sapiens*, *Sus scrofa*, *Ovis aries*, *Equus caballus* and *Balaenoptera acutorostrata*) retrieved from the NCBI database were aligned to CEY_v1 using tBLASTn (E-value ≤ 1e^−5^). GeneWise (v2.4.0)^37^ was then used to align against the matching proteins for accurate spliced alignments for the prediction of gene structure. Finally, GLEAN (v1.0.1)^38^ was used to combine homology with *de novo* gene models to form a comprehensive and non-redundant reference gene set with the following parameters: the minimum coding sequence length was 150 bp and maximum intron length was 10 Kb. We identified 20,652 protein-coding genes (Fig. 2 and Table 4) in our CEY_v1.

**Table 4:** The statistics of gene models of protein-coding genes annotated in the Tarim red deer genome.

Methods	Gene set	Number of genes	Average length (bp)				Exons per gene
			Gene length	CDS length	Exon length	Intron length	
*Ab initio*	Augustus	25,176	44,593.56	1,427.27	175.37	6,046.70	8.14
Homolog	*Bos taurus*	26,515	23,126.00	1,524.78	181.24	2,913.94	8.41
	*Canis familiaris*	28,410	40,491.39	1,575.50	180.72	5,042.44	8.72
	*Homo sapiens*	102,682	31,718.82	1,081.93	165.30	5,525.07	6.55
	*Ovis aries*	27,407	33,288.38	1,459.59	179.88	4,474.00	8.11
	*Sus scrofa*	29,486	23,673.50	1,267.90	184.48	3,815.11	6.87
	*Balaenoptera acutorostrata*	36,502	47,716.59	1,749.88	168.55	4,899.43	10.38
Glean		20,652	37,290.72	1,577.53	190.74	4,912.07	8.54

Functional annotation of the protein-coding genes was carried out using BLAST tools (E-value ≤ 1e^−5^) against the NCBI non-redundant proteins (NR), TrEMBL, Gene Ontology (GO), SwissProt^39^ and Kyoto Encyclopedia of Genes and Genomes (KEGG)^40^ respectively. Overall, 20,652 (100%) protein-coding genes were annotated with at least one public functional database (Table 5).

**Table 5:** Statistics of functional annotation.

Type	Number of overall predicted genes	Percentage of overall predicted genes
Total	20,652	100%
SwissProt	20,189	97.71%
KEGG	18,017	87.20%
TrEMBL	20,528	99.35%
NR	20,505	99.24%
GO	13,867	67.11%

## Data Records

Illumina DNA sequencing data from 10x Genomics libraries (Experiments under the SRA study accession: SRP220754) were submitted to the NCBI Sequence Read Archive (SRA) database under BioProject accession number PRJNA564362^41^. The assembled genome^42^ was deposited at DDBJ/ENA/GenBank under the accession WMHW00000000. The version described in this paper is version WMHW00000000.1^43^. Chromosome Y sequences of CEY_v1 were deposited at figshare^44^. Gene structure annotation, repeat predictions and gene functional annotation files of CEY_v1 were deposited at figshare^45^.

## Technical Validation

By comparing the assembled metrics of the scaffolds of Tarim red deer and the other deer species (Table 6), our CEY_v1 represents a substantial improvement in both contig and scaffold lengths, indicating that our assembly was highly contiguous. The similarity of the assembled length and the low number of gaps provide evidence that our CEY_v1 is a high quality genome assembly, which can be used with confidence for further downstream relevant analysis and investigation.

**Table 6:** Comparison of the deer genome assembly metrics.

Species	Assembled genome size (ungaped) (Gb)	Genome coverage (×)	Contig N50 (Kb)	Scaffold N50 (Mb)	Number of scaffolds
Tarim red deer (*Cervus elaphus yarkandensis*)	2.60 (2.56)	63	275.5	31.7	19,010
White-lipped deer (*Przewalskium albirostris*)^15^	2.69 (2.64)	214	39.6	3.8	171,874
Chinese water deer (*Hydropotes inermis*)^15^	2.53 (2.48)	76	131.4	13.8	22,246
Black muntjac (*Muntiacus crinifrons*)^15^	2.68 (2.67)	116	8.2	1.3	21,052
Hog deer (*Axis porcinus*)^17^	2.68 (2.64)	197	172.8	20.6	136,093
Milu (*Elaphurus davidianus*)^18^	2.52 (2.46)	82	32.7	3.0	46 381
Red deer (*Cervus elaphus*)^19^	3.40 (1.95)	62	7.9	0.27	34,724
Reeves muntjac (*Muntiacus reevesi*)^20^	2.58(2.51)	34	225.1	9.4	29,705
Muntjak (*Muntiacus muntjak*)^20^	2.57(2.52)	41	215.5	-	25,651
Mule deer (*Odocoileus hemionus*)^22^	2.34 (2.34)	25	113.3	0.8	838,758
Reindeer (*Rangifer tarandus*)^23^	2.64 (2.54)	220	89.7	0.94	58 765
Eastern roe deer (*Capreolus pygargu*)^24^	2.61 (2,55)	77	-	6.6	92,100
White-tailed deer (*Odocoileus virginianus*)^25^	2.38 (2.36)	150	122.0	0.9	17,025
Alces alces (*Eurasian elk*)^26^	2,74 (2,54)	35	131,8	4.1	48,219

**Fig. 3:**
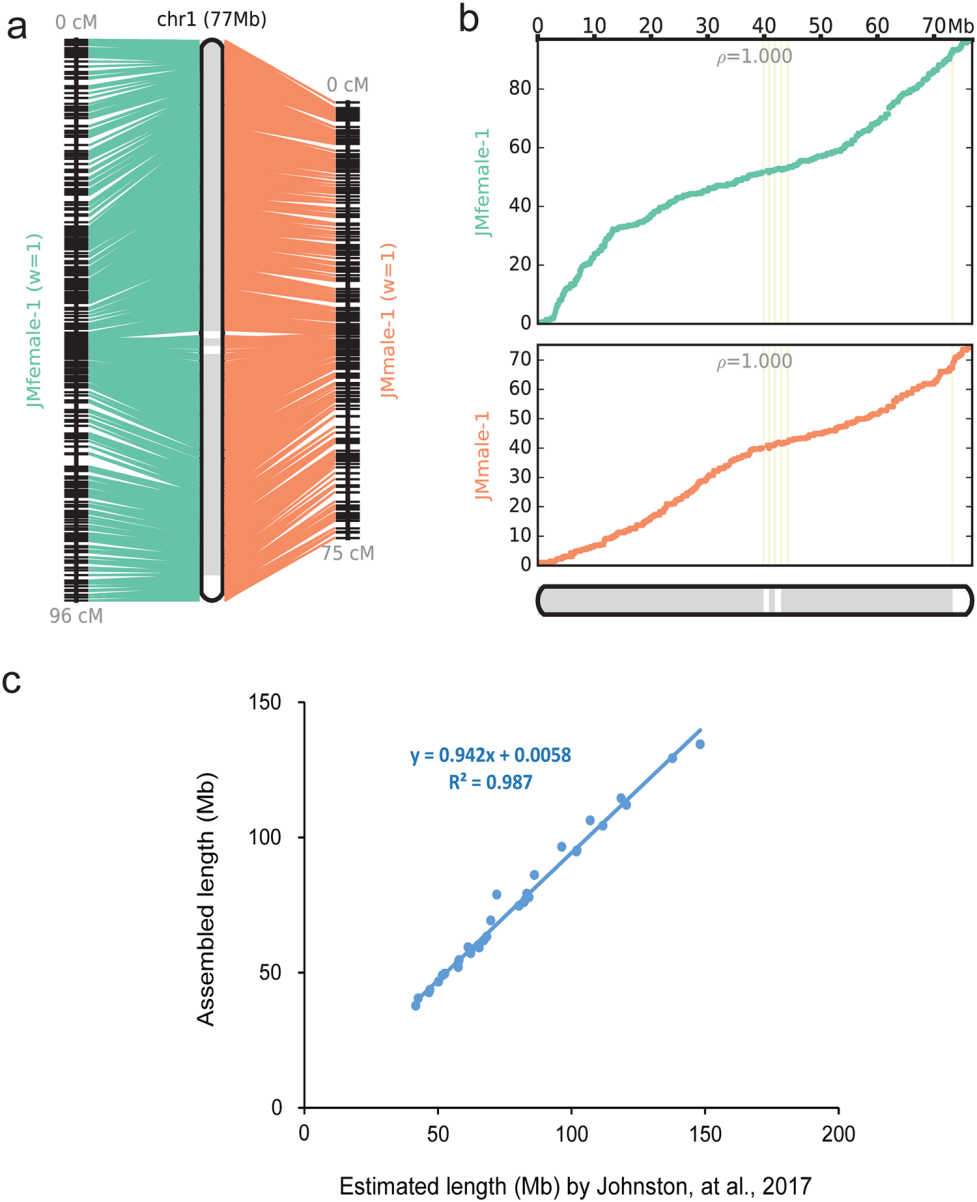
Reconstructed chromosome 1 of the Tarim red deer genome (CEY_v1) using two genetic maps: the red deer female and male genetic maps with equal weights. (**a**) “Side-by-side” alignments between chromosomes and the linkage groups. The conflict markers are shown as across lines. (**b**) Two scatter plots, in which dots representing the physical position (x-axis) versus the genetic map distance (y-axis) on the chromosomes, showed a monotonic trend and no breaks for illustrating near-perfect collinearity. Adjacent scaffolds within the chromosome are shown as boxes with alternation shades, marking the boundaries of the component scaffolds. The ρ-value on each scatter plot measures the Pearson correlation coefficient, with values in the range of −1 to 1 (values closer to −1 and 1 indicate near-perfect collinearity). (**c**) Correlation between the size of the reconstructed chromosomes and those of the previous estimation by Johnston, *et al*.^27^.

To estimate the quality of anchored chromosomes, we compared the physical and genetic maps. The reconstructed chromosomes showed few conflicting markers, and the female and male genetic maps exhibited perfect collinearity, except for chromosome X (i.e. chromosome 34) (Fig. 3a and Supplementary Fig. S1). Furthermore, two scatter plots, where dots represent the physical position (x-axis) versus the genetic map distance (y-axis), revealed no breaks, illustrating near-perfect collinearity (Fig. 3b and Supplementary Fig. S1). In addition, the size of the reconstructed chromosomes was highly consistent (R^2^ = 0.987) with previous estimates^27^, also indicating the high quality of anchorage of scaffolds onto chromosomes (Fig. 3c).

To assess the completeness of our CEY_v1, we performed an analysis using Benchmarking Universal Single-Copy Orthologs (BUSCO, v3.0) with the mammalia_odb9 database^46^. Our analysis showed that 94.1% of the expected mammalian genes (including 90.5% single and 3.6% duplicated ones) had complete gene coverage, and 2.3% were identified as fragmented, respectively, while 3.6% were considered missing in our CEY_v1.

## Supplementary information

Supplementary Fig S1

## Data Availability

No specific code was developed in this work. The data analyses were performed according to the manuals and protocols provided by the developers of the corresponding bioinformatics tools that are described in the Methods section together with the versions used.
