# Supplementary material for: Chromosome-level genome assembly of Tarim red deer, Cervus elaphus yarkandensis
Source: Sci Data. 2020 Jun 19;7:187. doi: 10.1038/s41597-020-0537-0 (PMC7305323; doi:10.1038/s41597-020-0537-0)
Supplement: Supplementary file 1 — Supplementary Fig S1 [file 41597_2020_537_MOESM1_ESM.pdf]

Fig. S1 Reconstructed 34 chromosomes of the Tarim red deer genome (CEY_v1) using the published high quality genetic linkage maps by Johnston, at al., 2017.

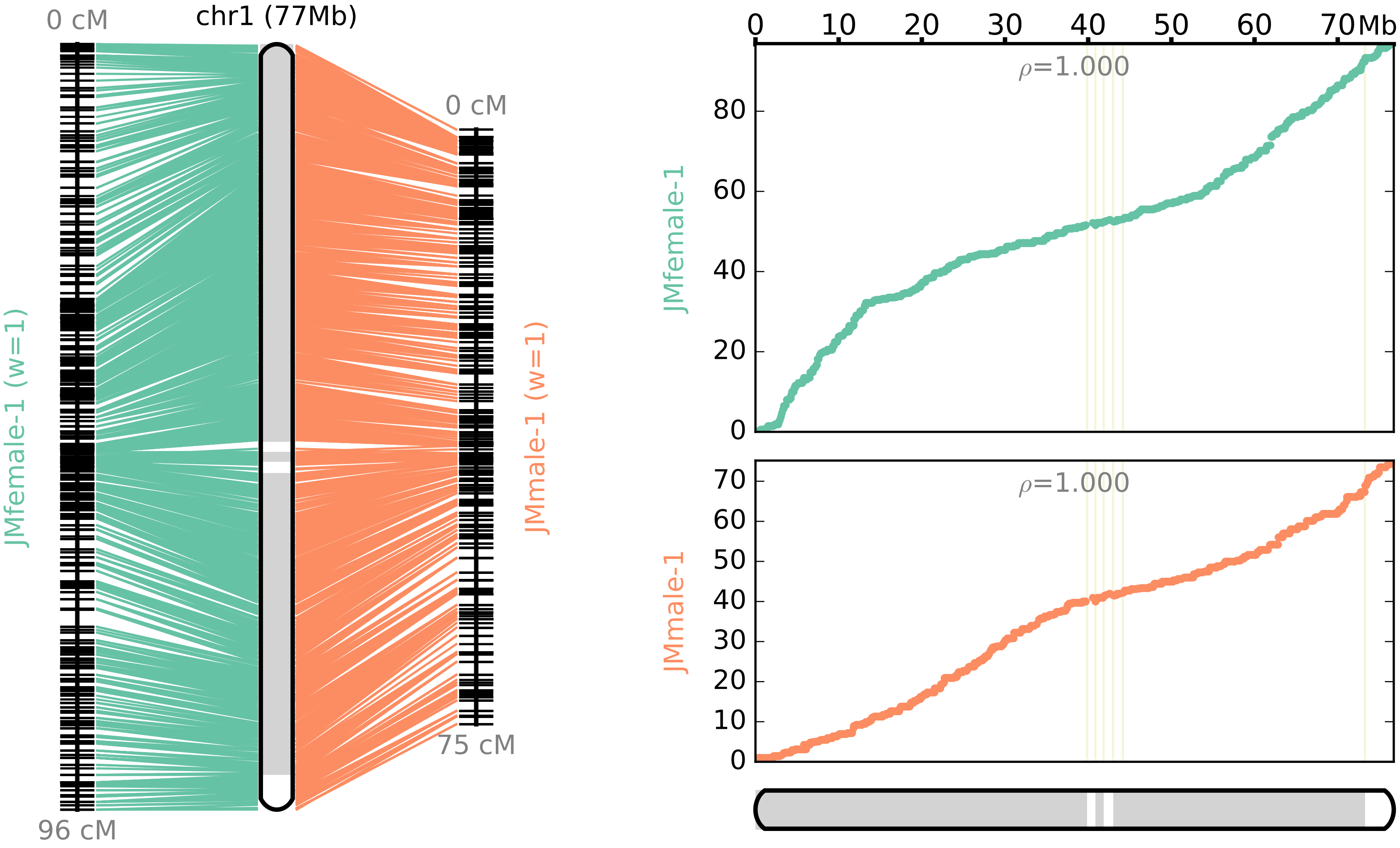

0 cM
chr1 (77Mb)
0 cM
JMfemale-1 (w=1)
JMmale-1 (w=1)
75 cM
96 cM
0
10
20
30
40
50
60
70Mb
ρ=1.000
80
60
40
20
0
JMfemale-1
ρ=1.000
70
60
50
40
30
20
10
0
JMmale-1

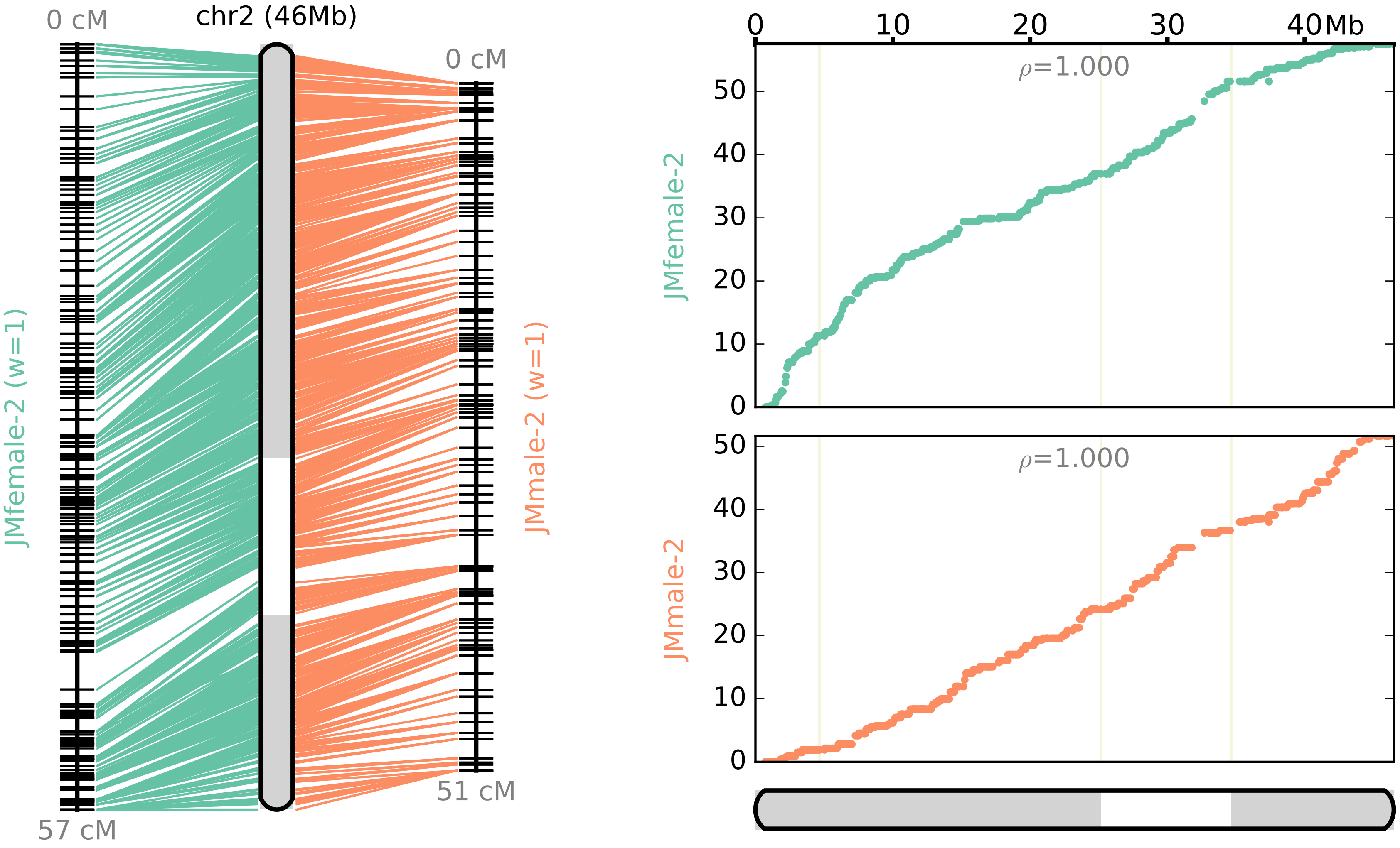

0 cM
chr2 (46Mb)
0 cM
JMfemale-2 (w=1)
JMmale-2 (w=1)
57 cM
51 cM
0
10
20
30
40Mb
ρ=1.000
JMfemale-2
50
40
30
20
10
0
ρ=1.000
JMmale-2
50
40
30
20
10
0

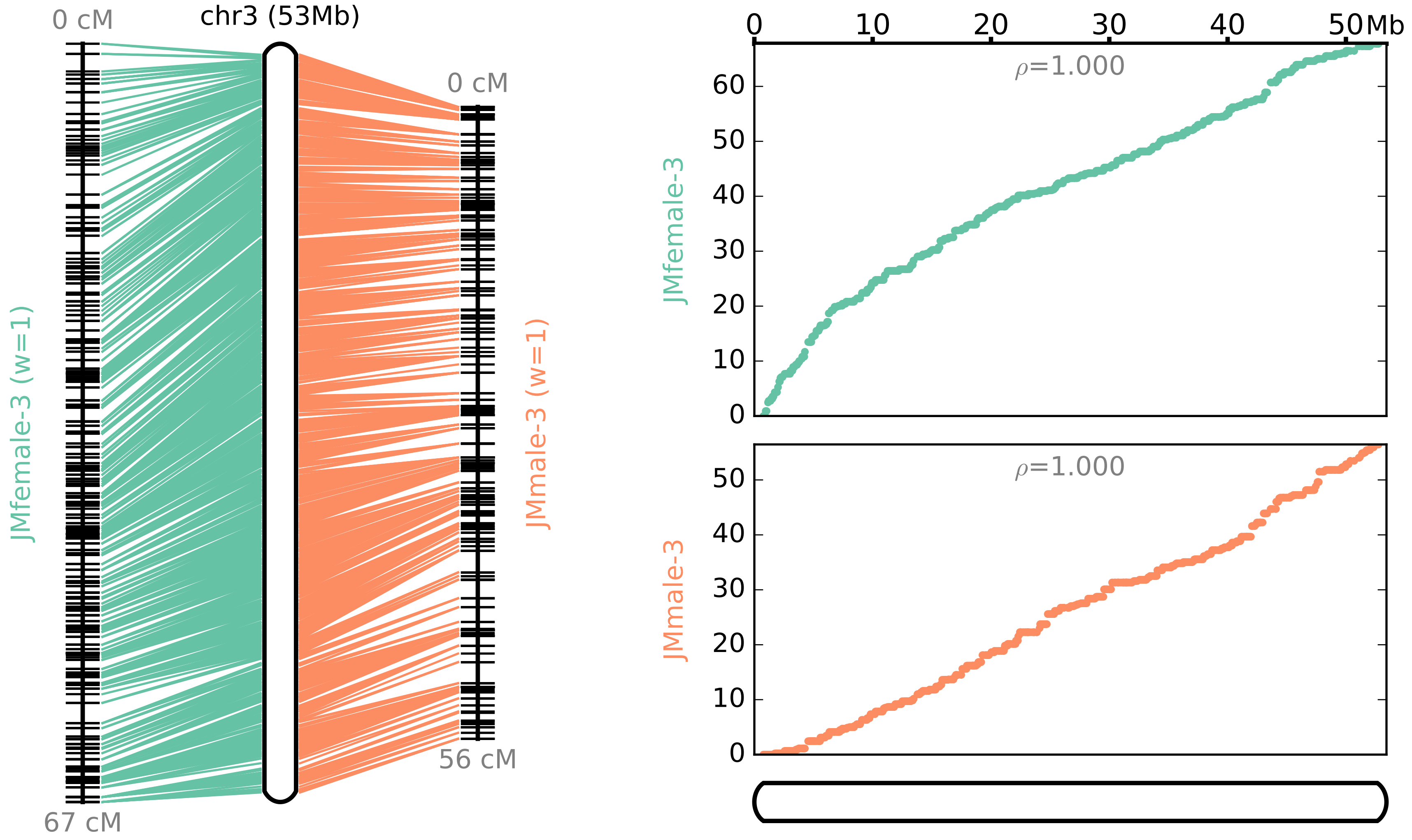
0 cM
chr3 (53Mb)
0 cM
JMfemale-3 (w=1)
JMmale-3 (w=1)
56 cM
67 cM
0
10
20
30
40
50Mb
ρ=1.000
JMfemale-3
60
50
40
30
20
10
0
ρ=1.000
JMmale-3
50
40
30
20
10
0

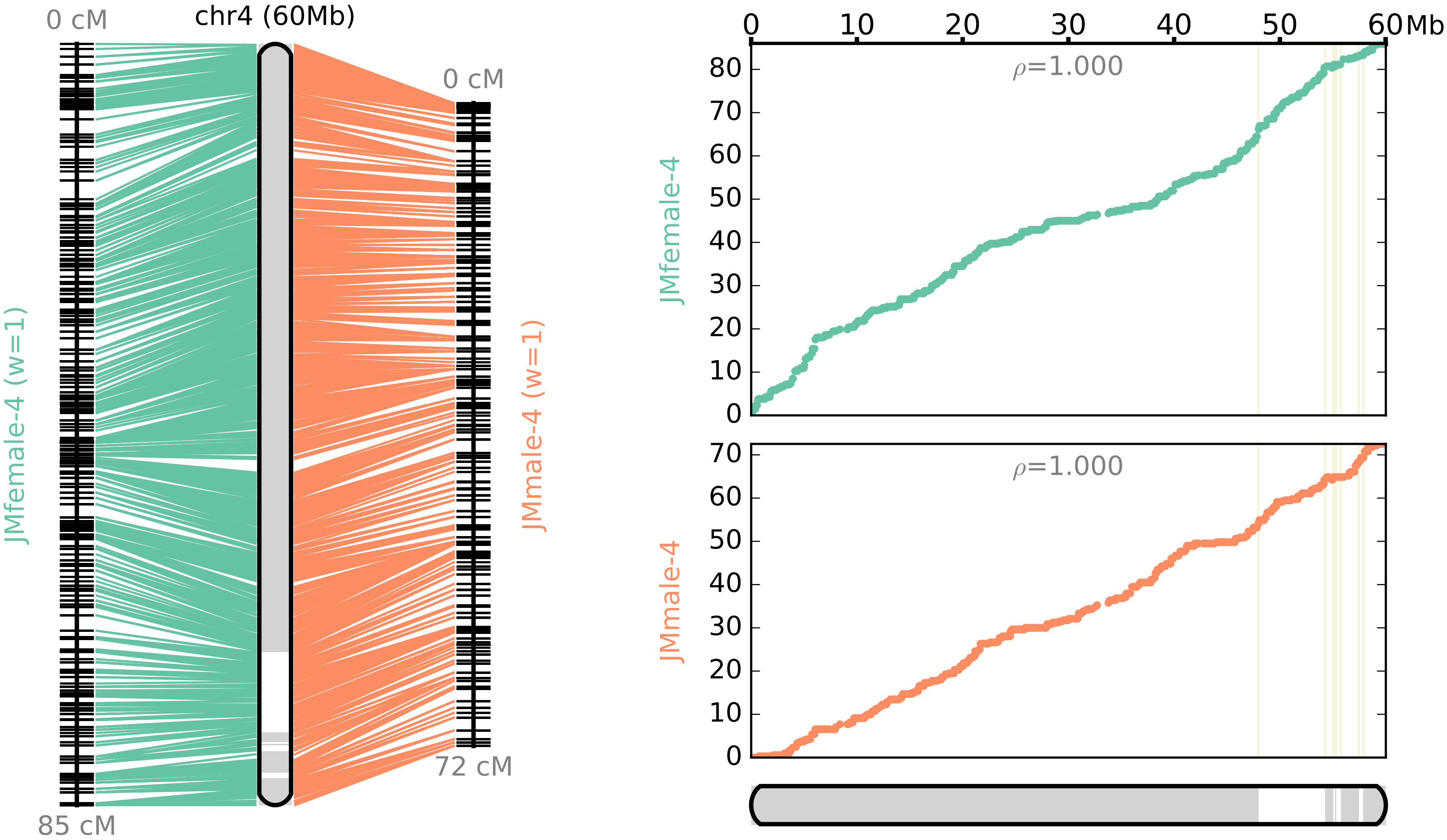

0 cM
chr4 (60Mb)
0 cM
JMfemale-4 (w=1)
JMmale-4 (w=1)
72 cM
85 cM
0 10 20 30 40 50 60Mb
ρ=1.000
JMfemale-4
0 10 20 30 40 50 60 70 80
ρ=1.000
JMmale-4
0 10 20 30 40 50 60 70

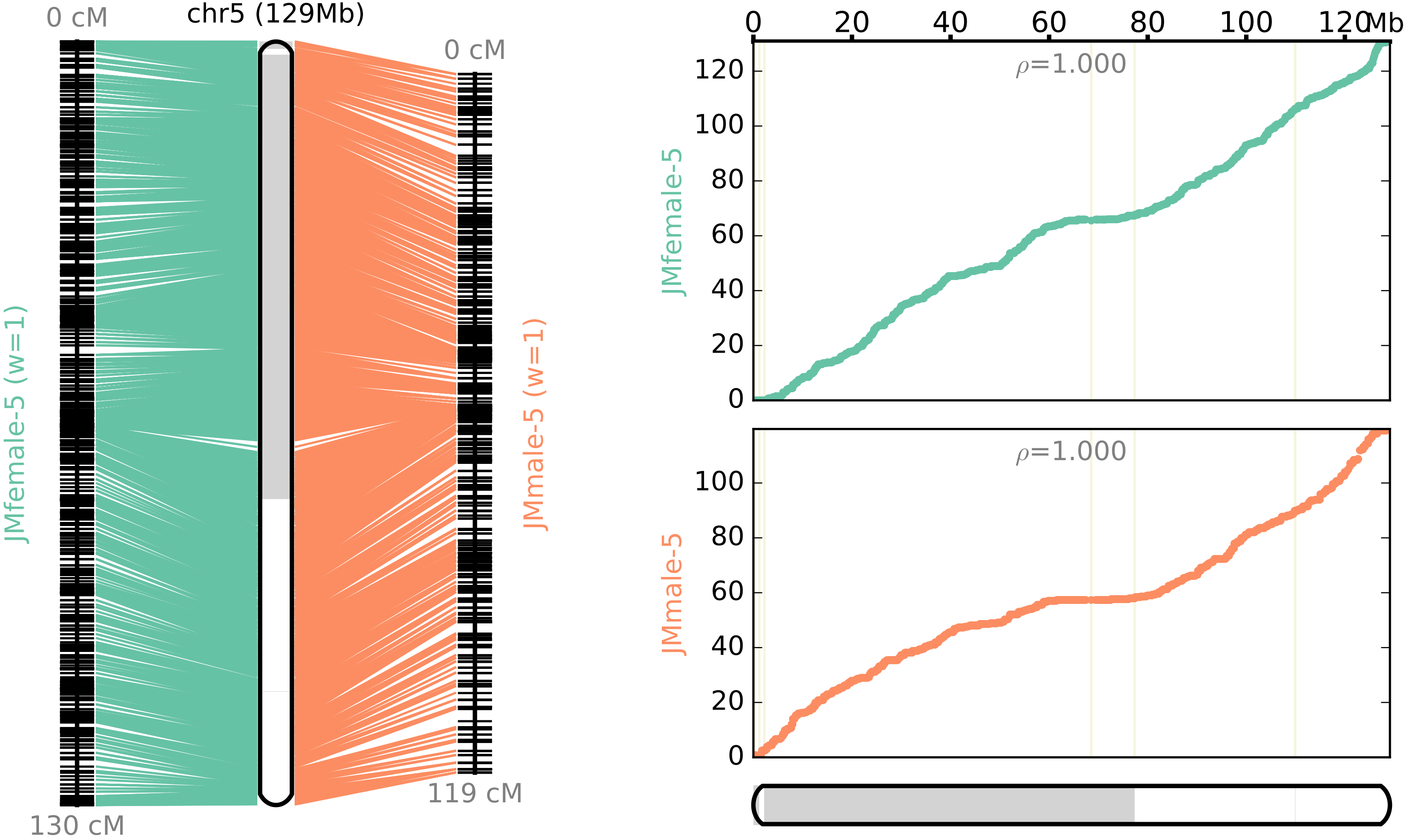

0 cM
chr5 (129Mb)
0 cM
JMfemale-5 (w=1)
JMmale-5 (w=1)
130 cM
119 cM
0
20
40
60
80
100
120
Mb
ρ=1.000
JMfemale-5
JMmale-5
ρ=1.000

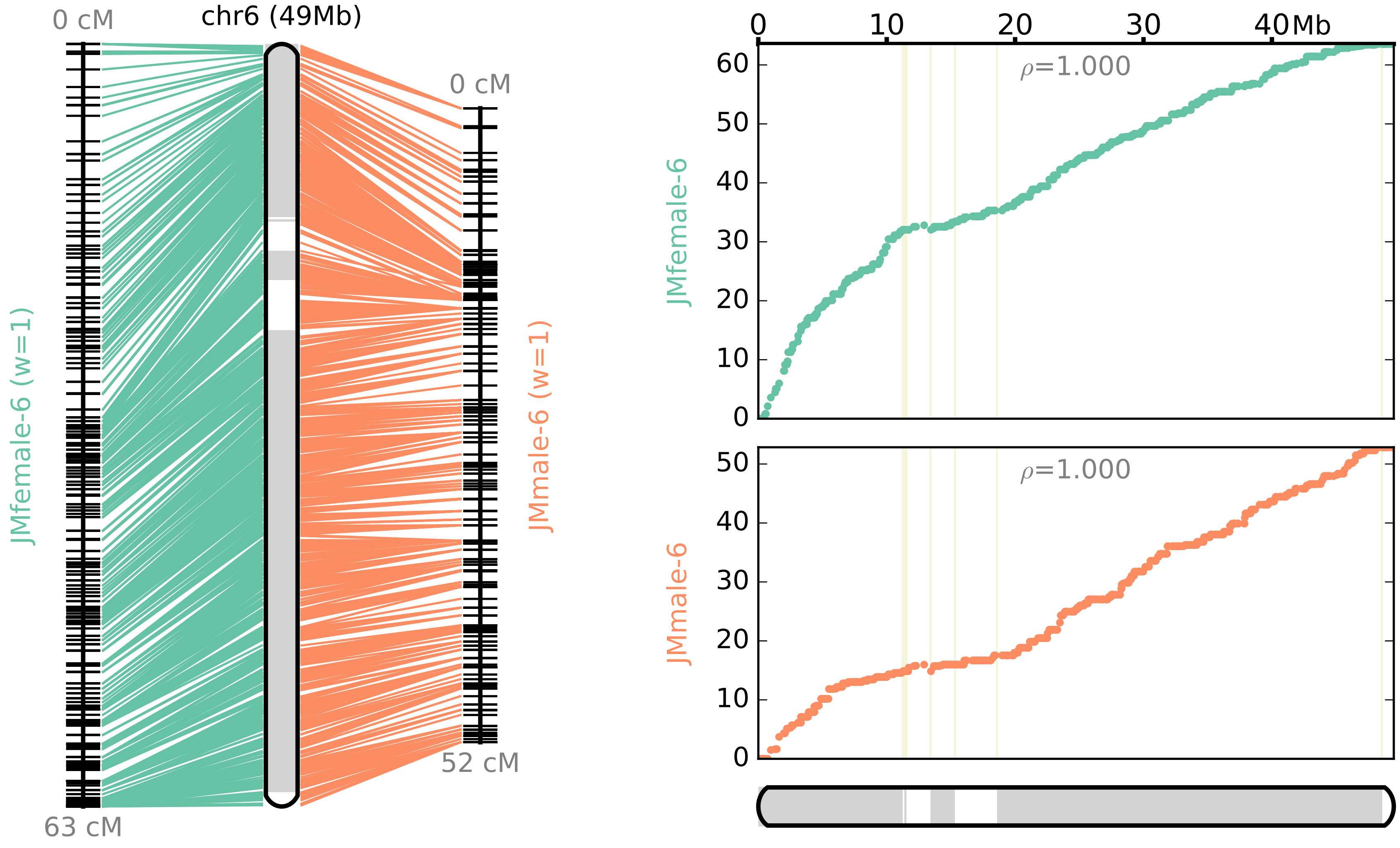

0 cM
chr6 (49Mb)
0 cM
JMfemale-6 (w=1)
JMmale-6 (w=1)
63 cM
52 cM
0
10
20
30
40Mb
ρ=1.000
JMfemale-6
60
50
40
30
20
10
0
ρ=1.000
JMmale-6
50
40
30
20
10
0

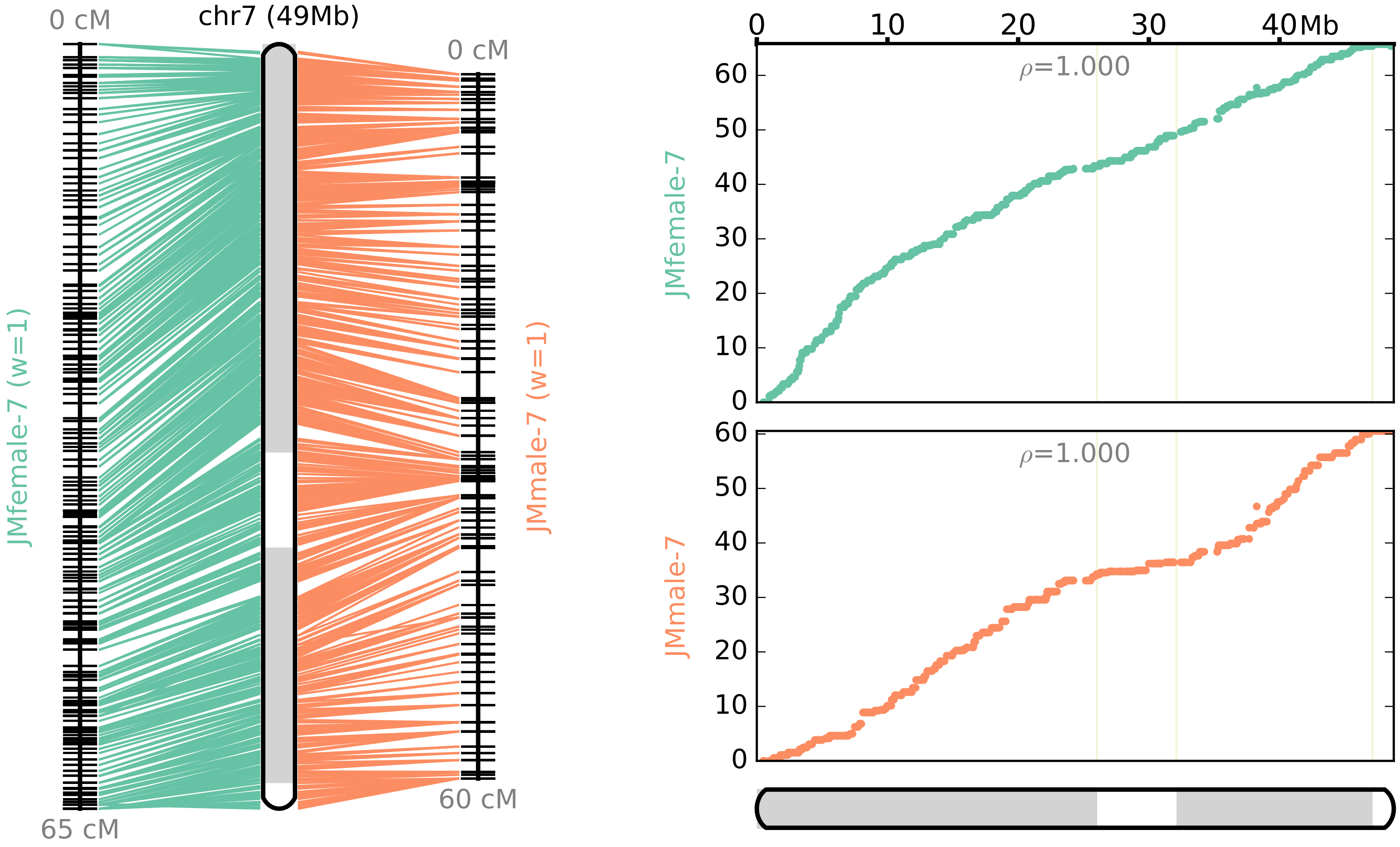

0 cM
chr7 (49Mb)
0 cM
JMfemale-7 (w=1)
JMmale-7 (w=1)
65 cM
60 cM
0
10
20
30
40Mb
ρ=1.000
JMfemale-7
JMmale-7

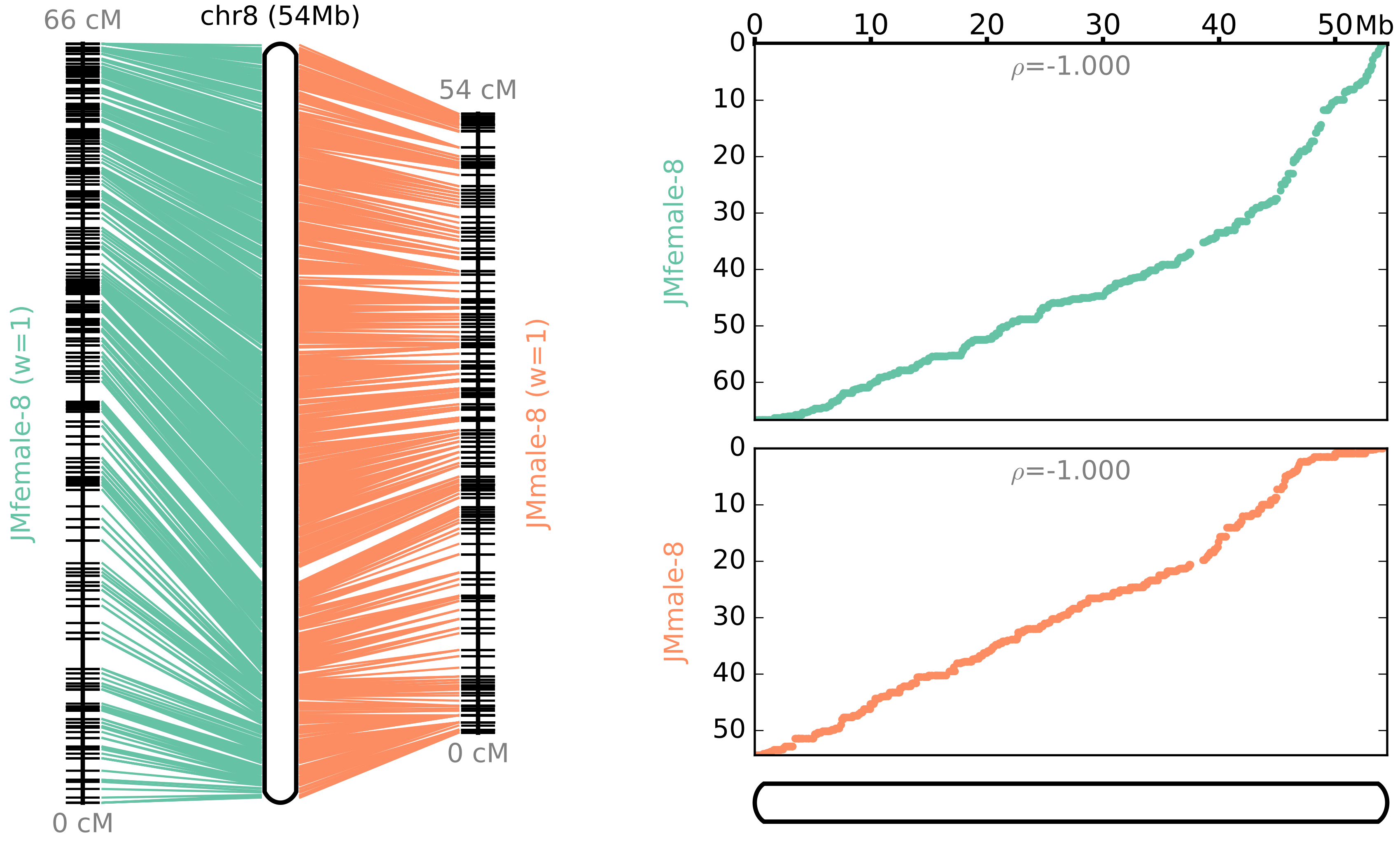

66 cM
chr8 (54Mb)
54 cM
0 cM
0 cM
JMfemale-8 (w=1)
JMmale-8 (w=1)
0
10
20
30
40
50Mb
ρ=-1.000
JMfemale-8
0
10
20
30
40
50
60
ρ=-1.000
JMmale-8
0
10
20
30
40
50

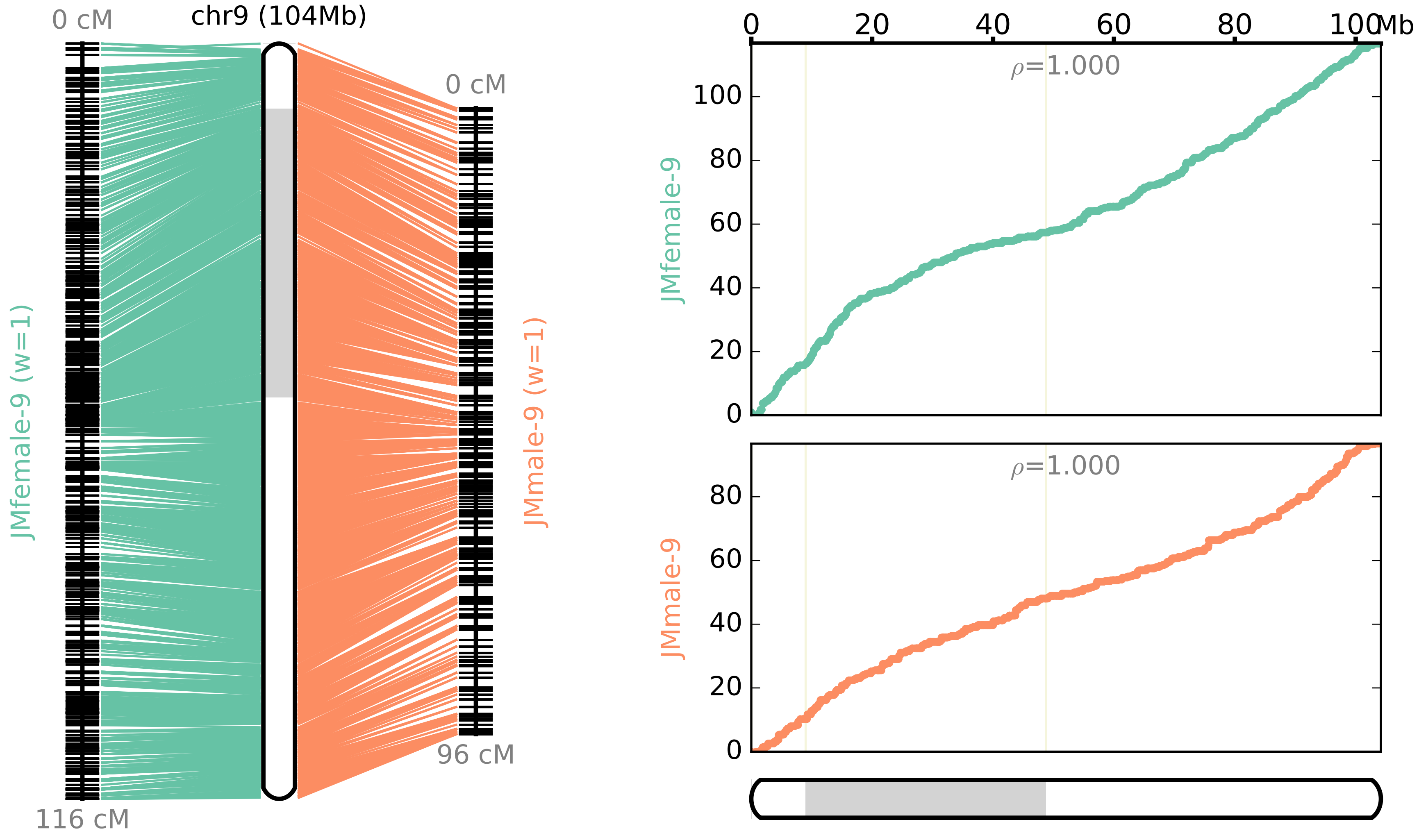

0 cM
chr9 (104Mb)
0 cM
JMfemale-9 (w=1)
JMmale-9 (w=1)
116 cM
96 cM
0
20
40
60
80
100
Mb
ρ=1.000
JMfemale-9
JMmale-9

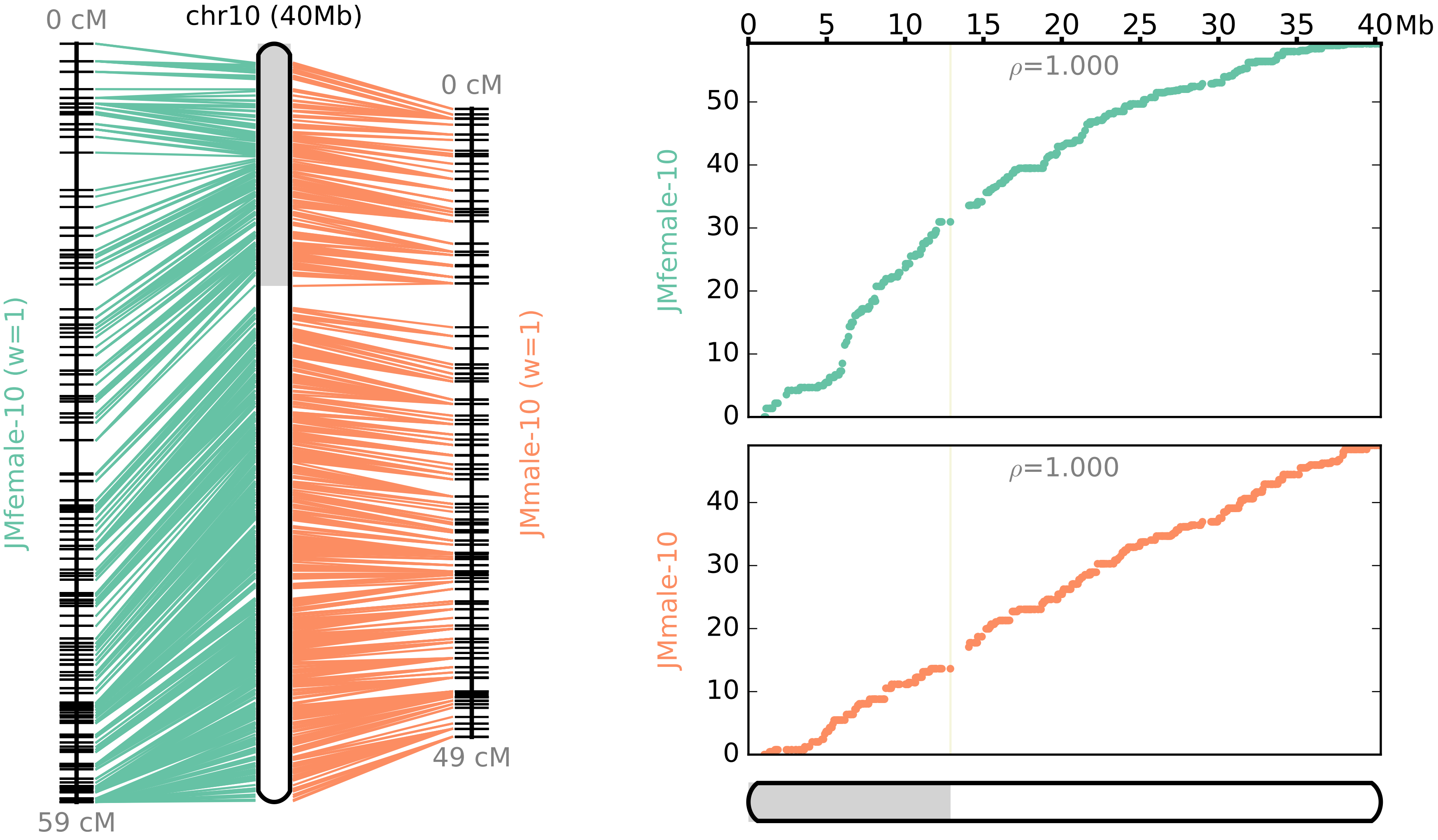

0 cM
chr10 (40Mb)
0 cM
49 cM
59 cM
JMfemale-10 (w=1)
JMmale-10 (w=1)
0
5
10
15
20
25
30
35
40Mb
ρ=1.000
JMfemale-10
0
10
20
30
40
50
ρ=1.000
JMmale-10
0
10
20
30
40

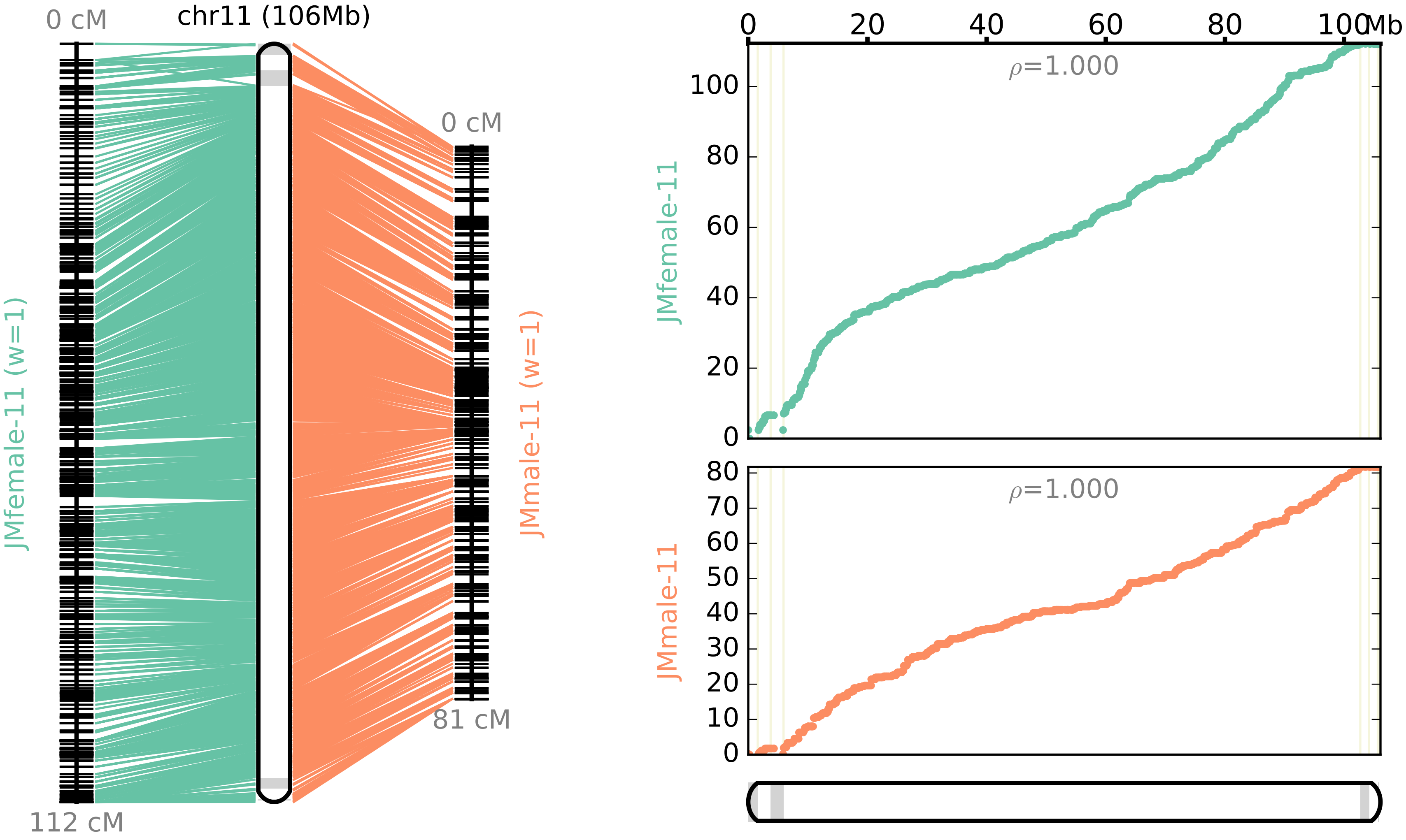

0 cM
chr11 (106Mb)
0 cM
112 cM
81 cM
JMfemale-11 (w=1)
JMmale-11 (w=1)
0
20
40
60
80
100
Mb
ρ=1.000
JMfemale-11
JMmale-11

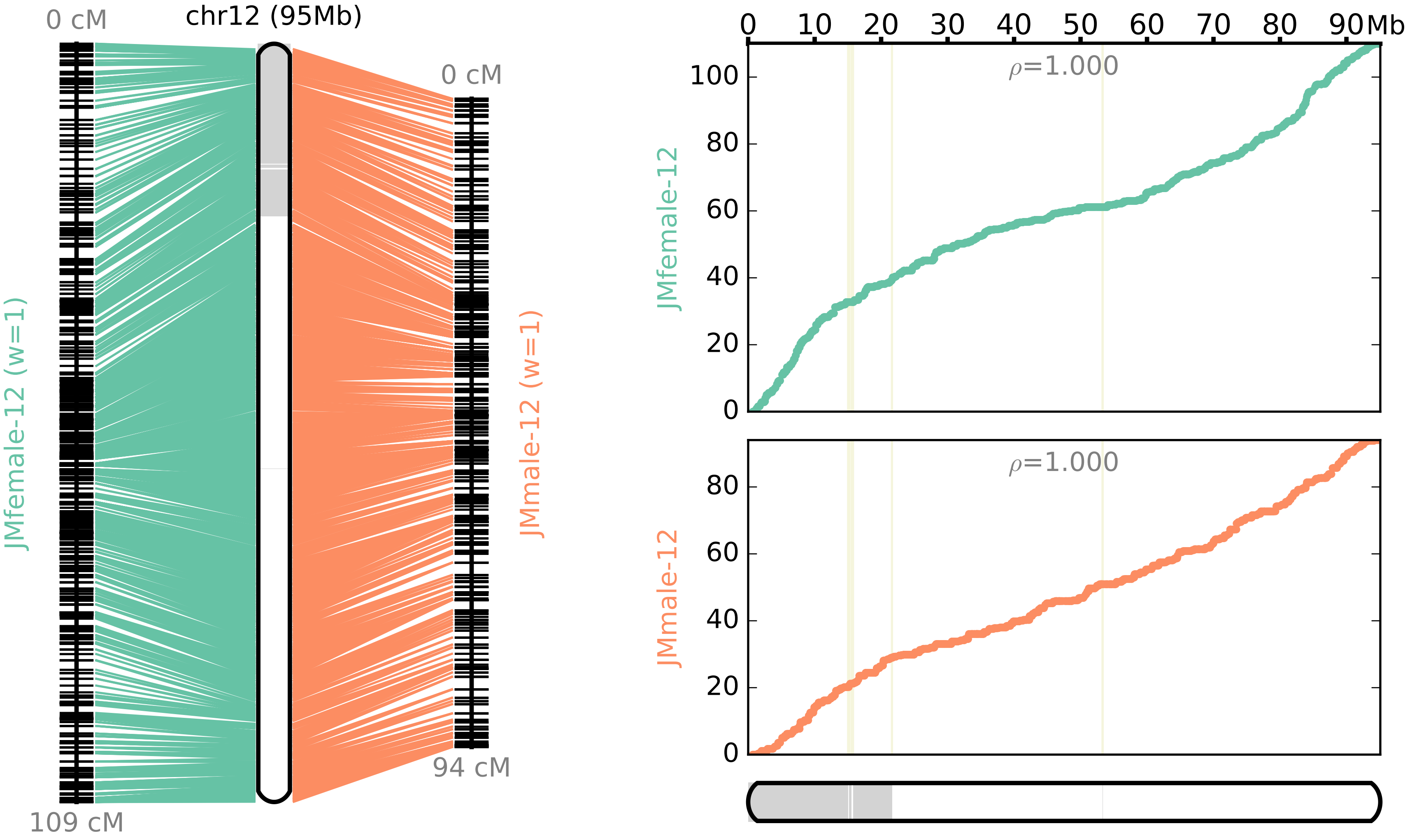

0 cM
chr12 (95Mb)
0 cM
JMfemale-12 (w=1)
JMmale-12 (w=1)
94 cM
109 cM
0 10 20 30 40 50 60 70 80 90Mb
ρ=1.000
JMfemale-12
0 20 40 60 80 100
ρ=1.000
JMmale-12
0 20 40 60 80

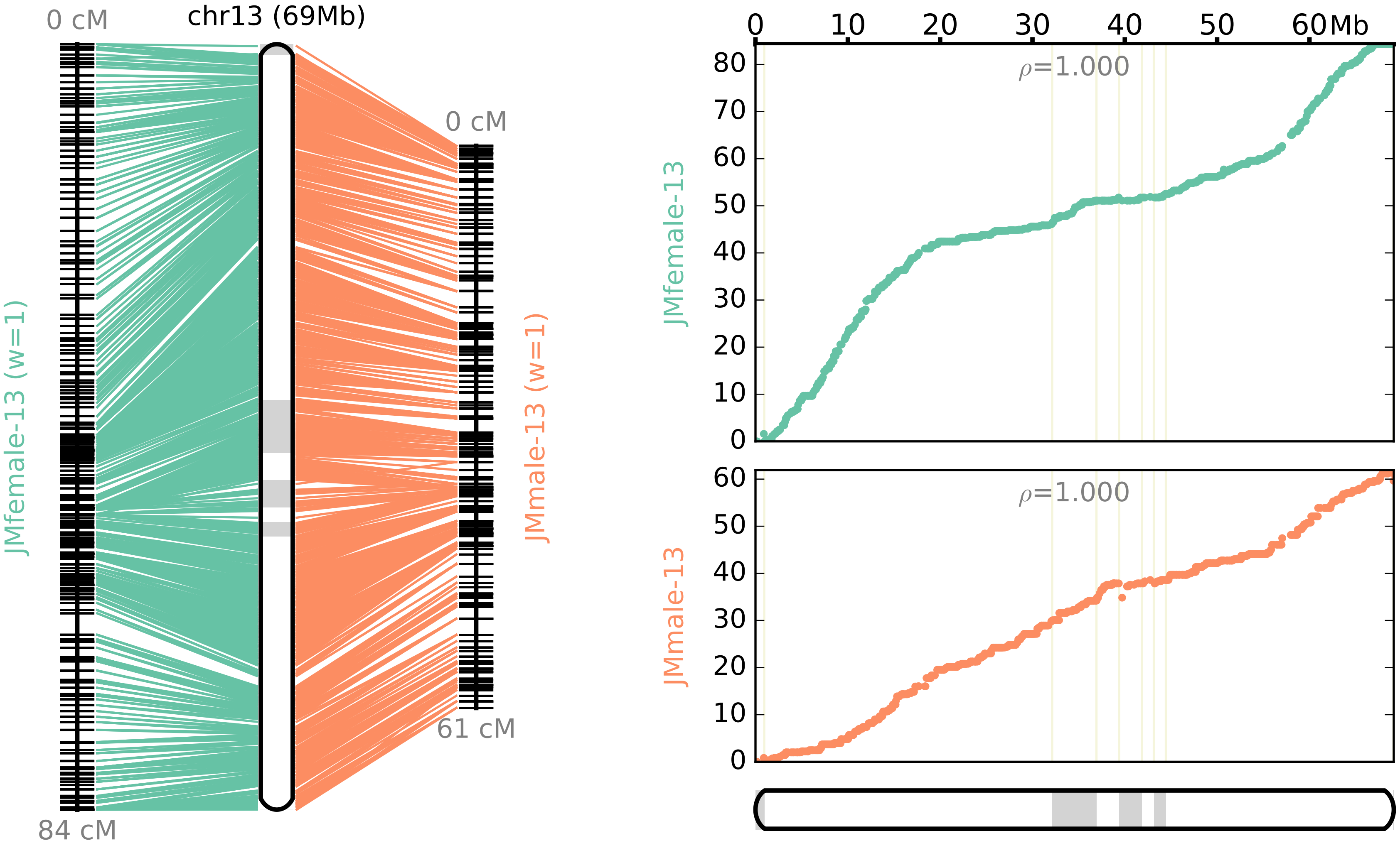
0 cM
chr13 (69Mb)
0 cM
JMfemale-13 (w=1)
JMmale-13 (w=1)
84 cM
61 cM
0
10
20
30
40
50
60Mb
80
70
60
50
40
30
20
10
0
ρ=1.000
JMfemale-13
60
50
40
30
20
10
0
ρ=1.000
JMmale-13

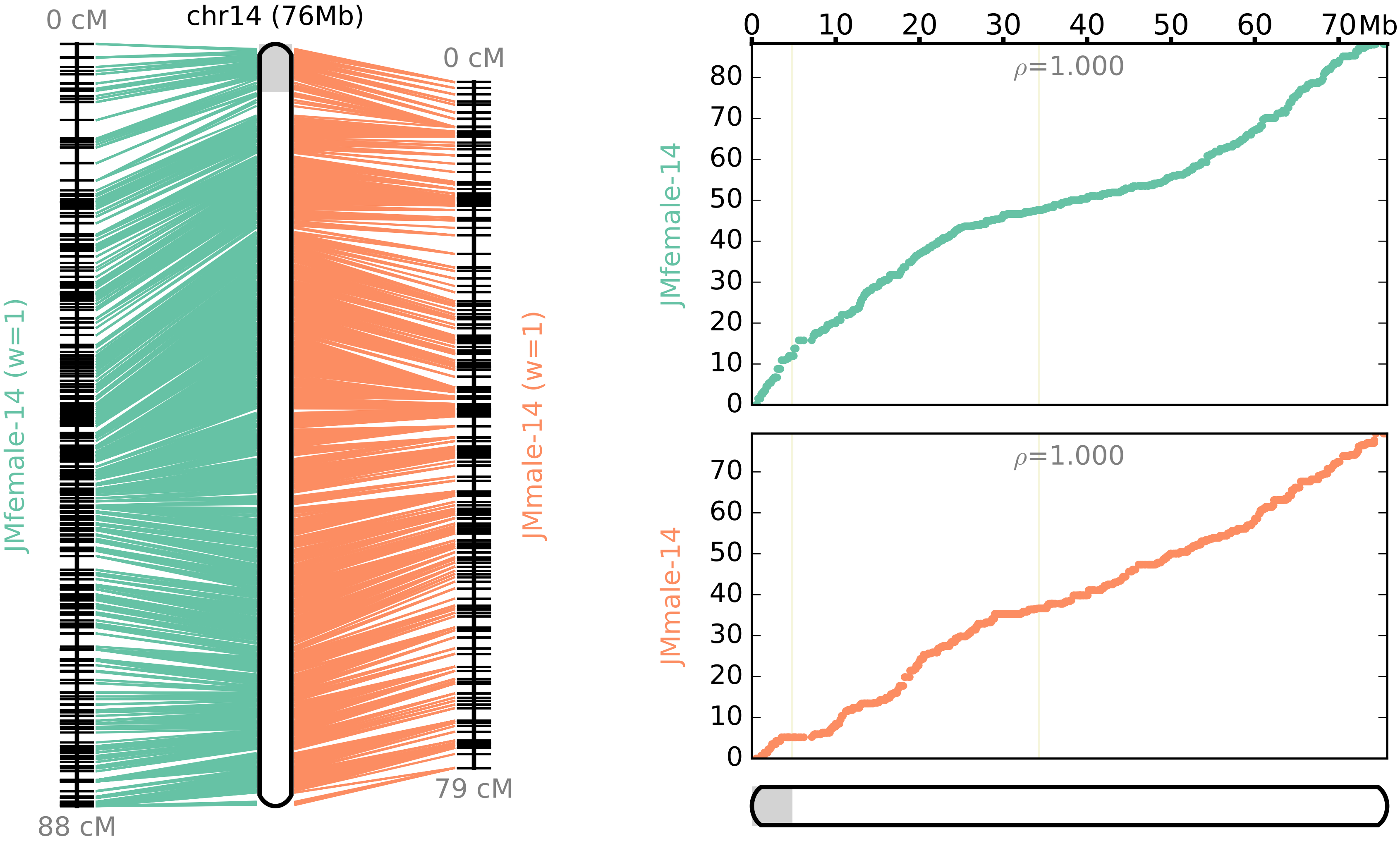

0 cM
chr14 (76Mb)
0 cM
JMfemale-14 (w=1)
JMmale-14 (w=1)
88 cM
79 cM
0 10 20 30 40 50 60 70Mb
JMfemale-14
ρ=1.000
0 10 20 30 40 50 60 70 80
JMmale-14
ρ=1.000
0 10 20 30 40 50 60 70

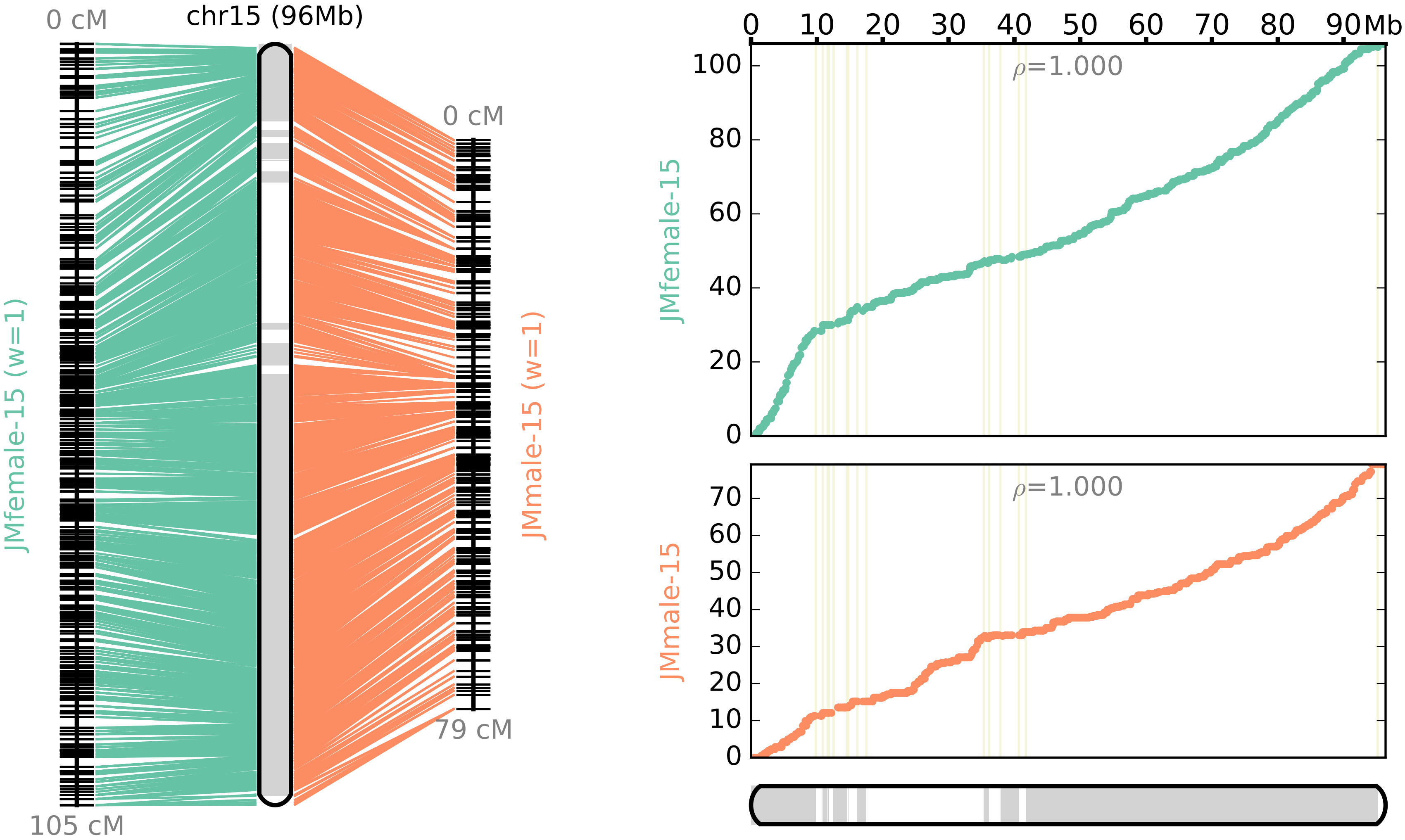

0 cM
chr15 (96Mb)
0 cM
JMfemale-15 (w=1)
JMmale-15 (w=1)
79 cM
105 cM
0 10 20 30 40 50 60 70 80 90Mb
JMfemale-15
ρ=1.000
0 20 40 60 80 100
JMmale-15
ρ=1.000
0 10 20 30 40 50 60 70

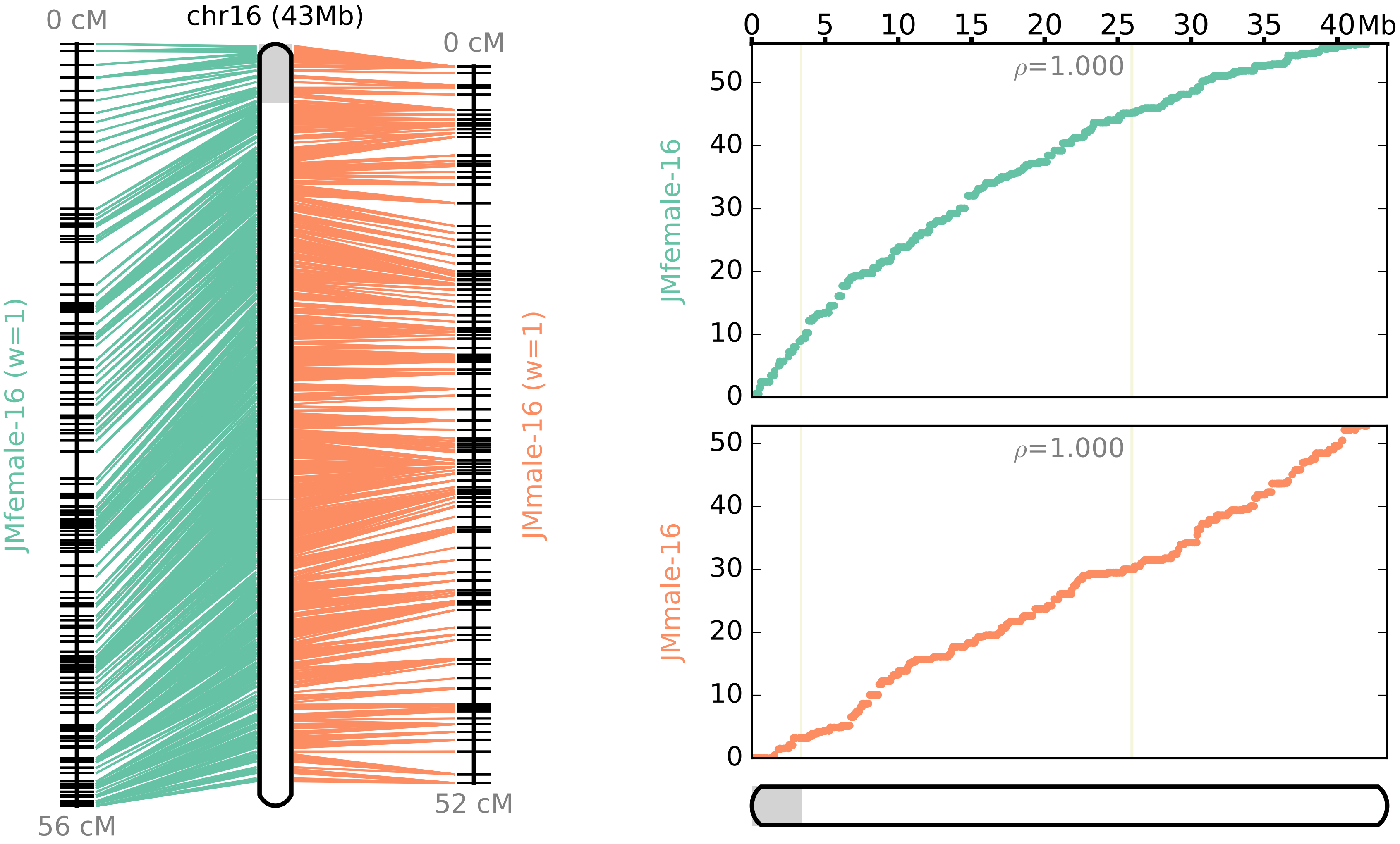

chr16 (43Mb)
0 cM
56 cM
0 cM
52 cM
JMfemale-16 (w=1)
JMmale-16 (w=1)
0 5 10 15 20 25 30 35 40Mb
JMfemale-16
ρ=1.000
JMmale-16
ρ=1.000

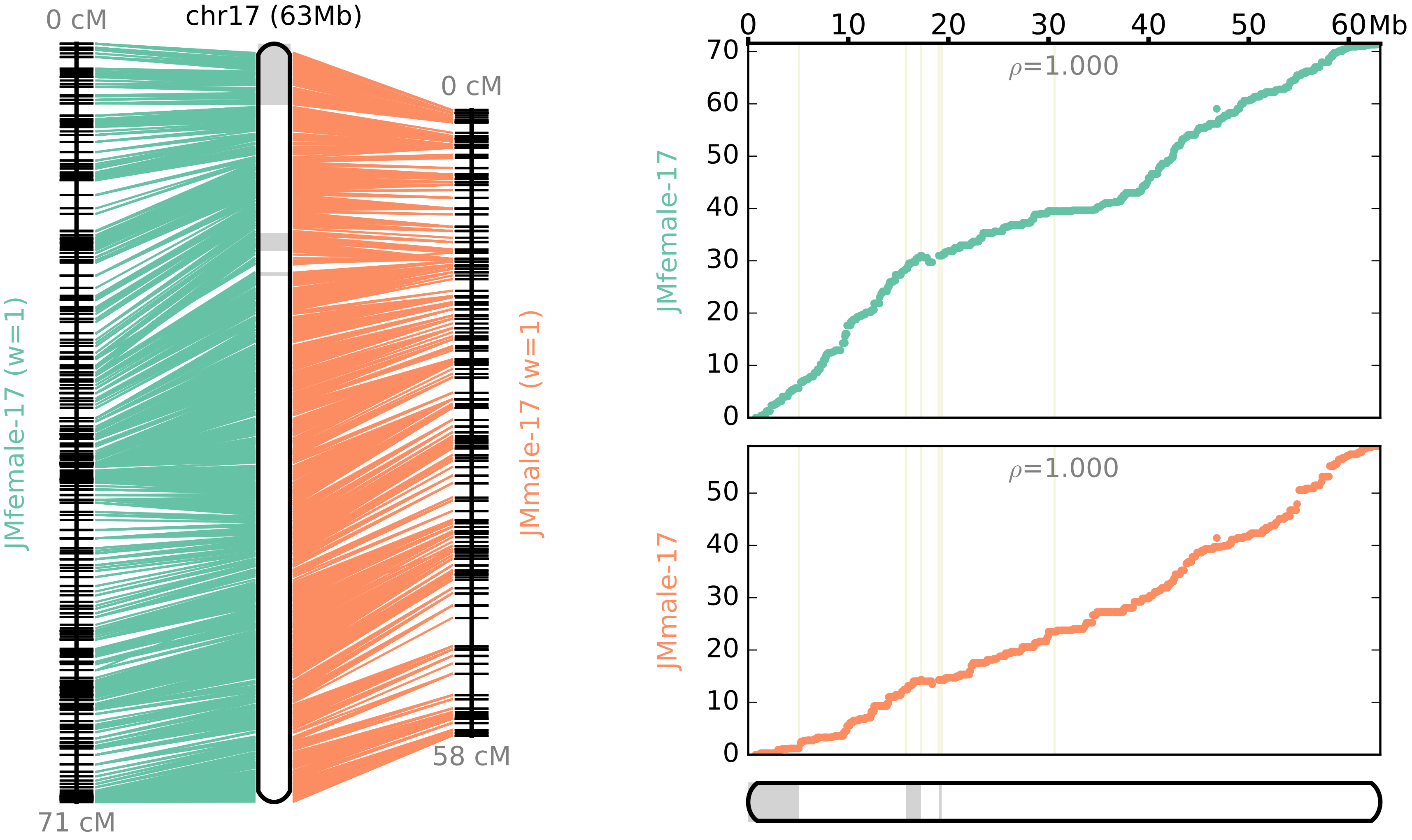

0 cM
chr17 (63Mb)
0 cM
71 cM
58 cM
JMfemale-17 (w=1)
JMmale-17 (w=1)
0
10
20
30
40
50
60Mb
ρ=1.000
JMfemale-17
JMmale-17

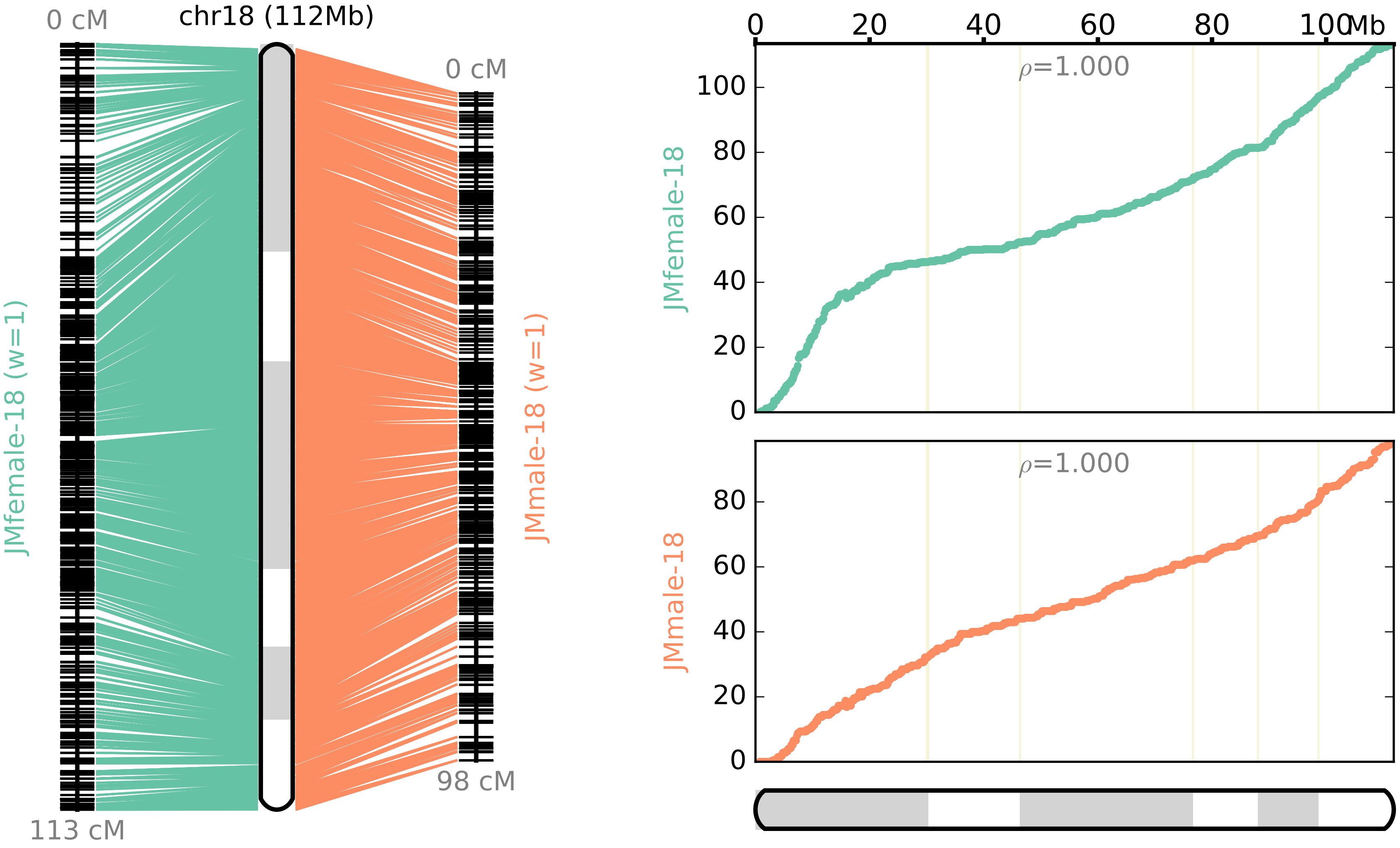

0 cM
chr18 (112Mb)
0 cM
JMfemale-18 (w=1)
JMmale-18 (w=1)
113 cM
98 cM
0
20
40
60
80
100
Mb
JMfemale-18
ρ=1.000
JMmale-18
ρ=1.000

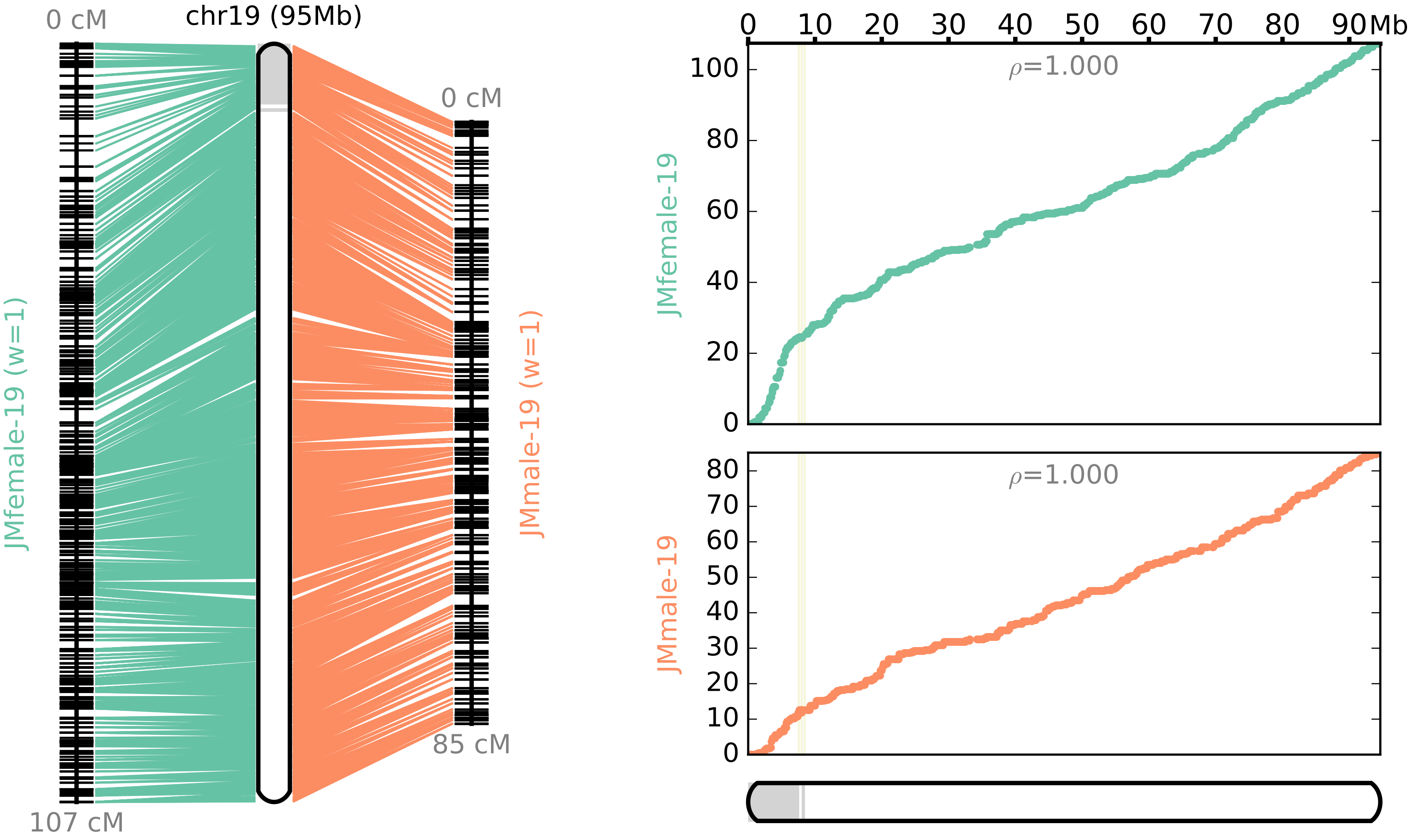

0 cM
chr19 (95Mb)
0 cM
JMfemale-19 (w=1)
JMmale-19 (w=1)
107 cM
85 cM
0
10
20
30
40
50
60
70
80
90Mb
ρ=1.000
JMfemale-19
JMmale-19
100
80
60
40
20
0
70
50
30
10

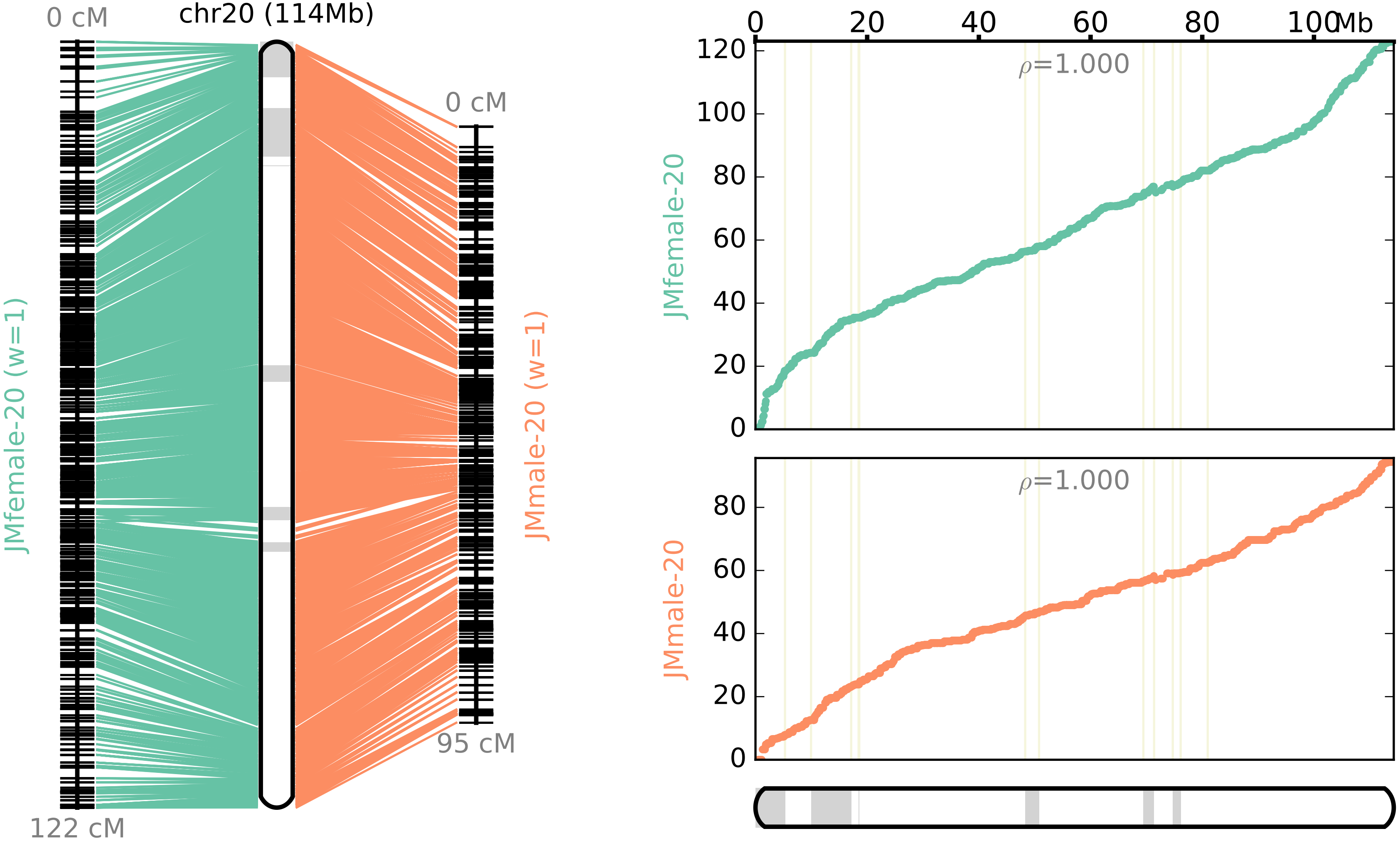

0 cM
chr20 (114Mb)
0 cM
95 cM
122 cM
JMfemale-20 (w=1)
JMmale-20 (w=1)
0
20
40
60
80
100
Mb
120
100
80
60
40
20
0
ρ=1.000
JMfemale-20
80
60
40
20
0
ρ=1.000
JMmale-20

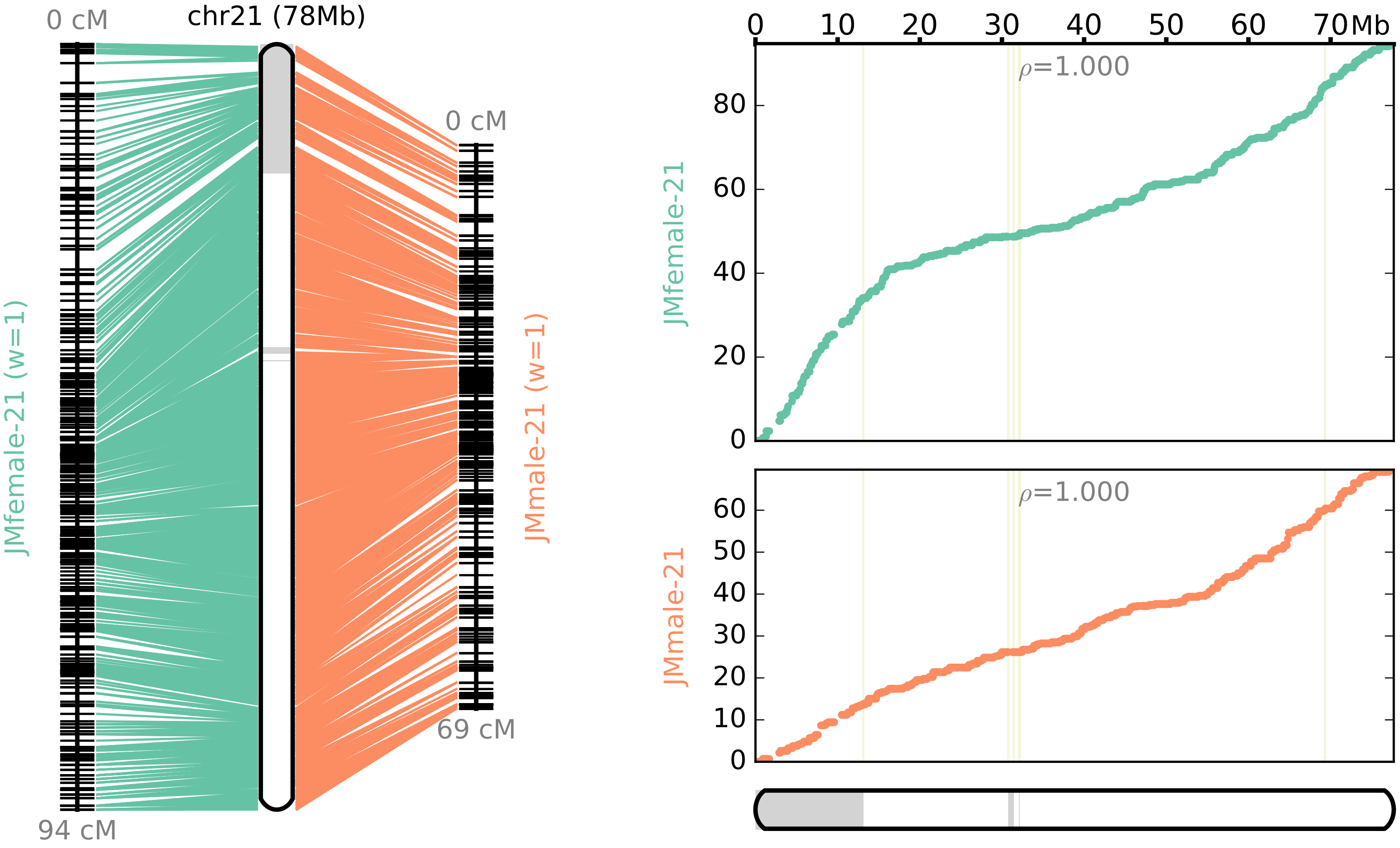

0 cM
chr21 (78Mb)
0 cM
94 cM
69 cM
JMfemale-21 (w=1)
JMmale-21 (w=1)
0
10
20
30
40
50
60
70Mb
ρ=1.000
JMfemale-21
0
20
40
60
80
ρ=1.000
JMmale-21
0
10
20
30
40
50
60

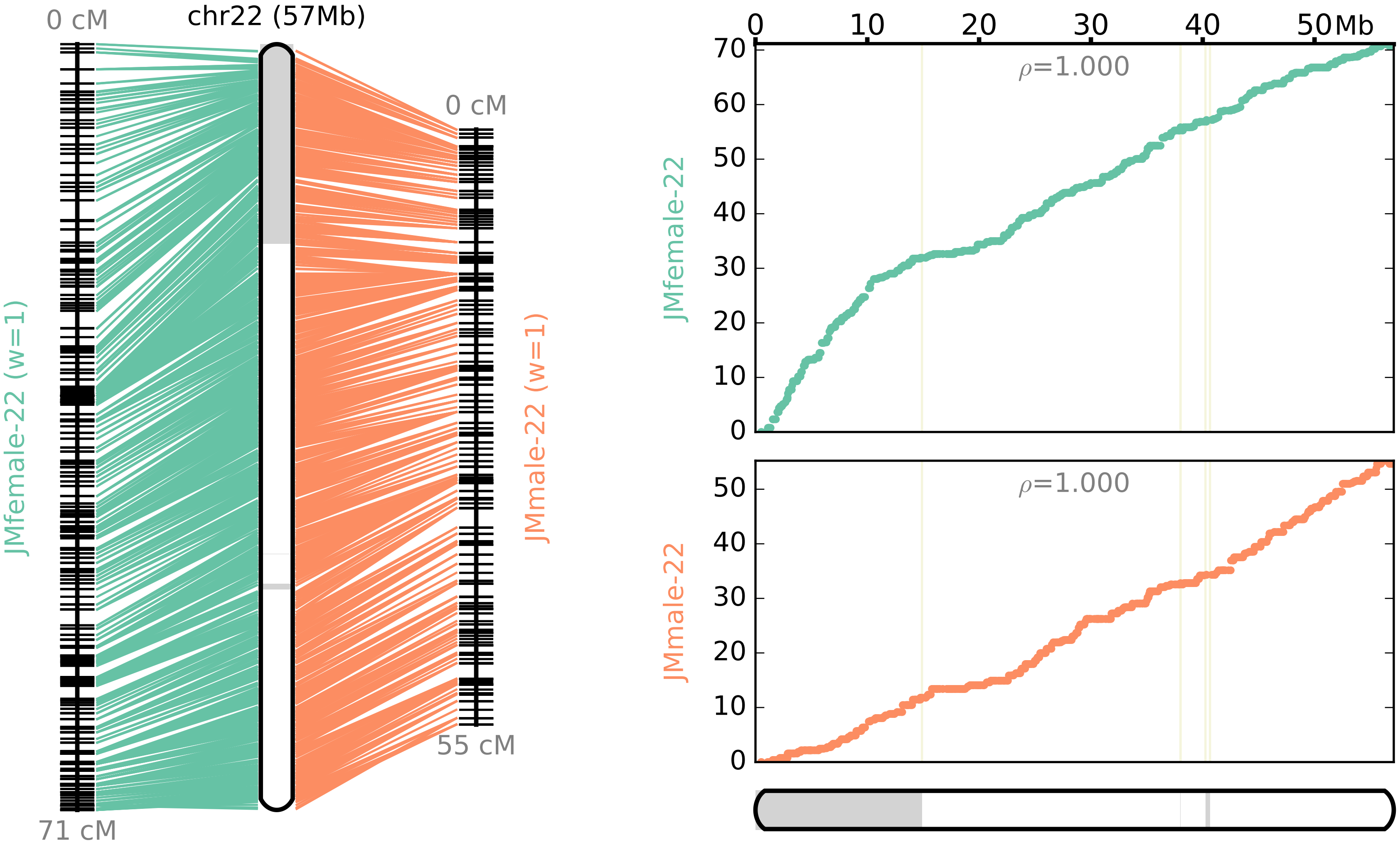

0 cM
chr22 (57Mb)
0 cM
JMfemale-22 (w=1)
JMmale-22 (w=1)
55 cM
71 cM
0
10
20
30
40
50Mb
70
60
50
40
30
20
10
0
ρ=1.000
JMfemale-22
50
40
30
20
10
0
ρ=1.000
JMmale-22

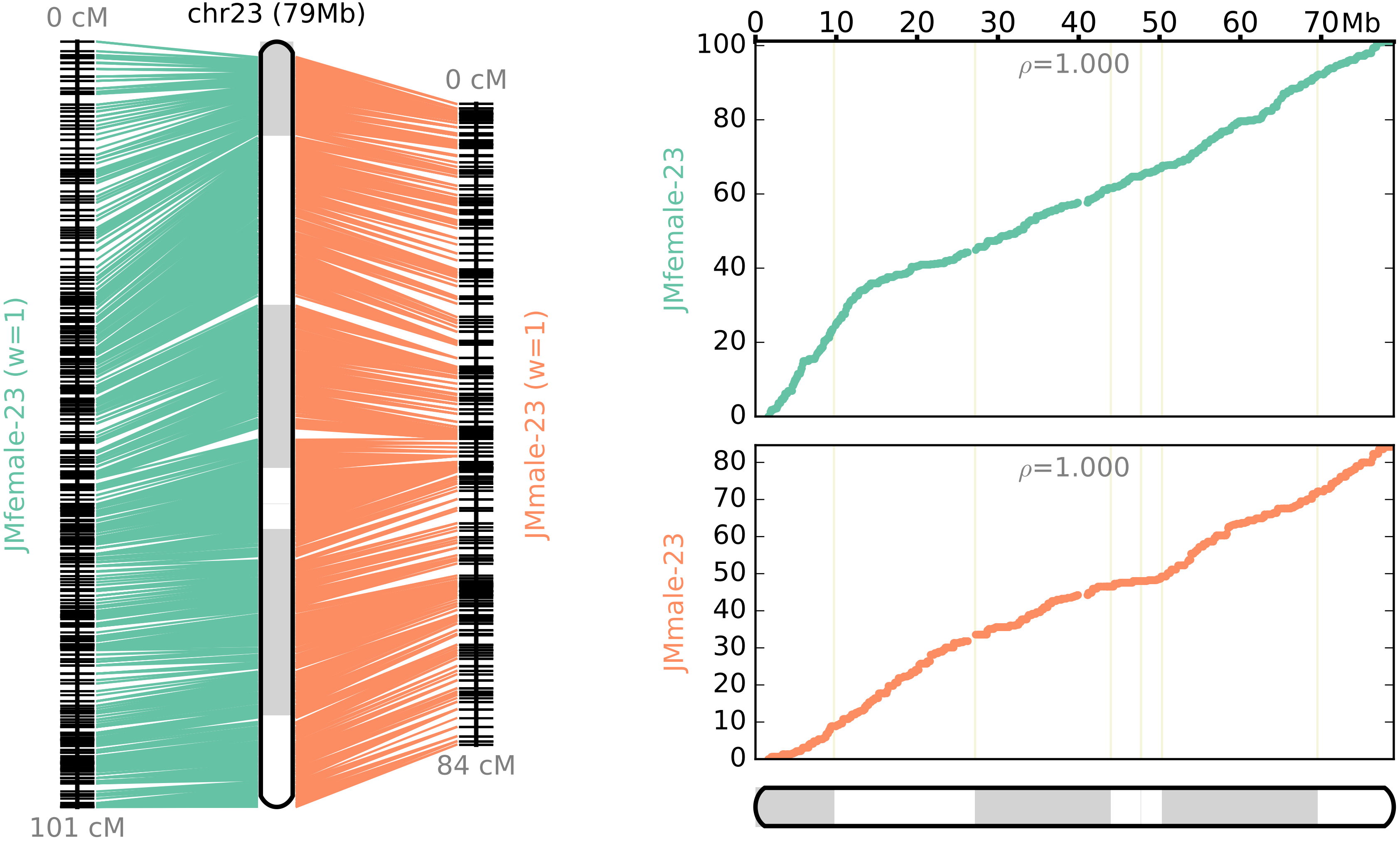

0 cM
chr23 (79Mb)
0 cM
101 cM
84 cM
JMfemale-23 (w=1)
JMmale-23 (w=1)
0
10
20
30
40
50
60
70Mb
100
80
60
40
20
0
ρ=1.000
JMfemale-23
80
70
60
50
40
30
20
10
0
ρ=1.000
JMmale-23

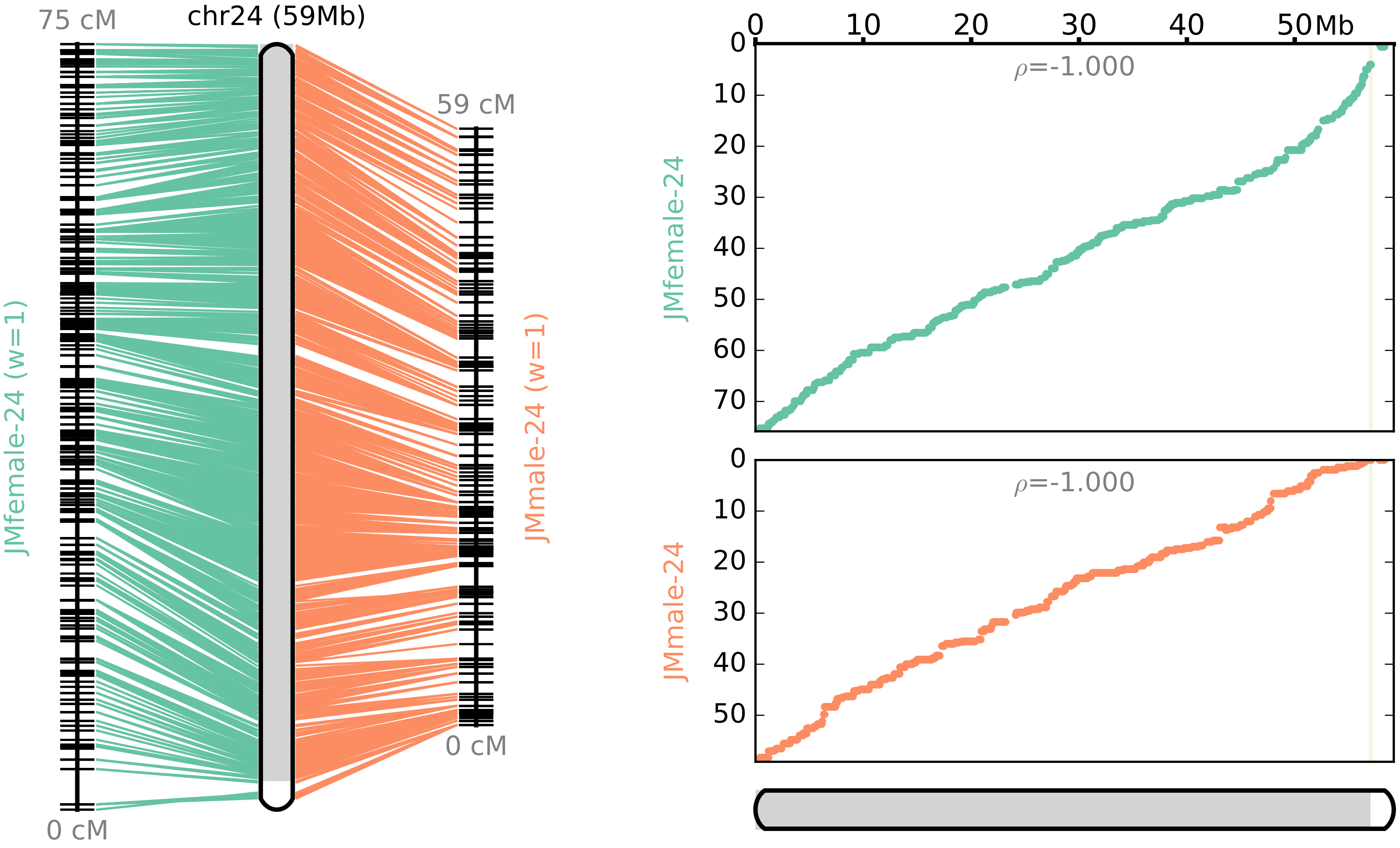

75 cM
chr24 (59Mb)
59 cM
0 cM
0 cM
JMfemale-24 (w=1)
JMmale-24 (w=1)
0
10
20
30
40
50Mb
ρ=-1.000
JMfemale-24
0
10
20
30
40
50
60
70
ρ=-1.000
JMmale-24
0
10
20
30
40
50

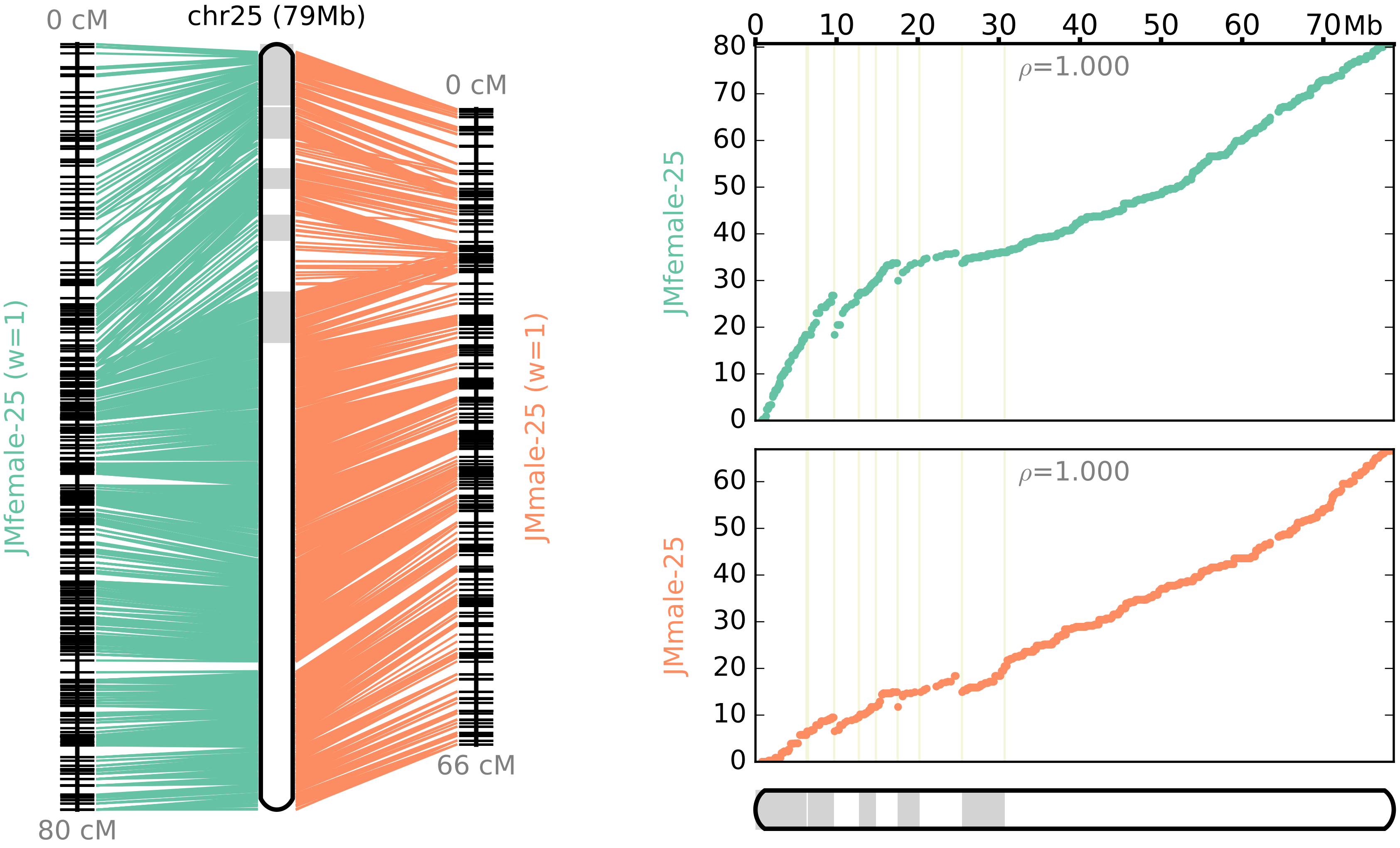

0 cM
chr25 (79Mb)
0 cM
JMfemale-25 (w=1)
JMmale-25 (w=1)
66 cM
80 cM
0 10 20 30 40 50 60 70Mb
JMfemale-25
JMmale-25
ρ=1.000
ρ=1.000

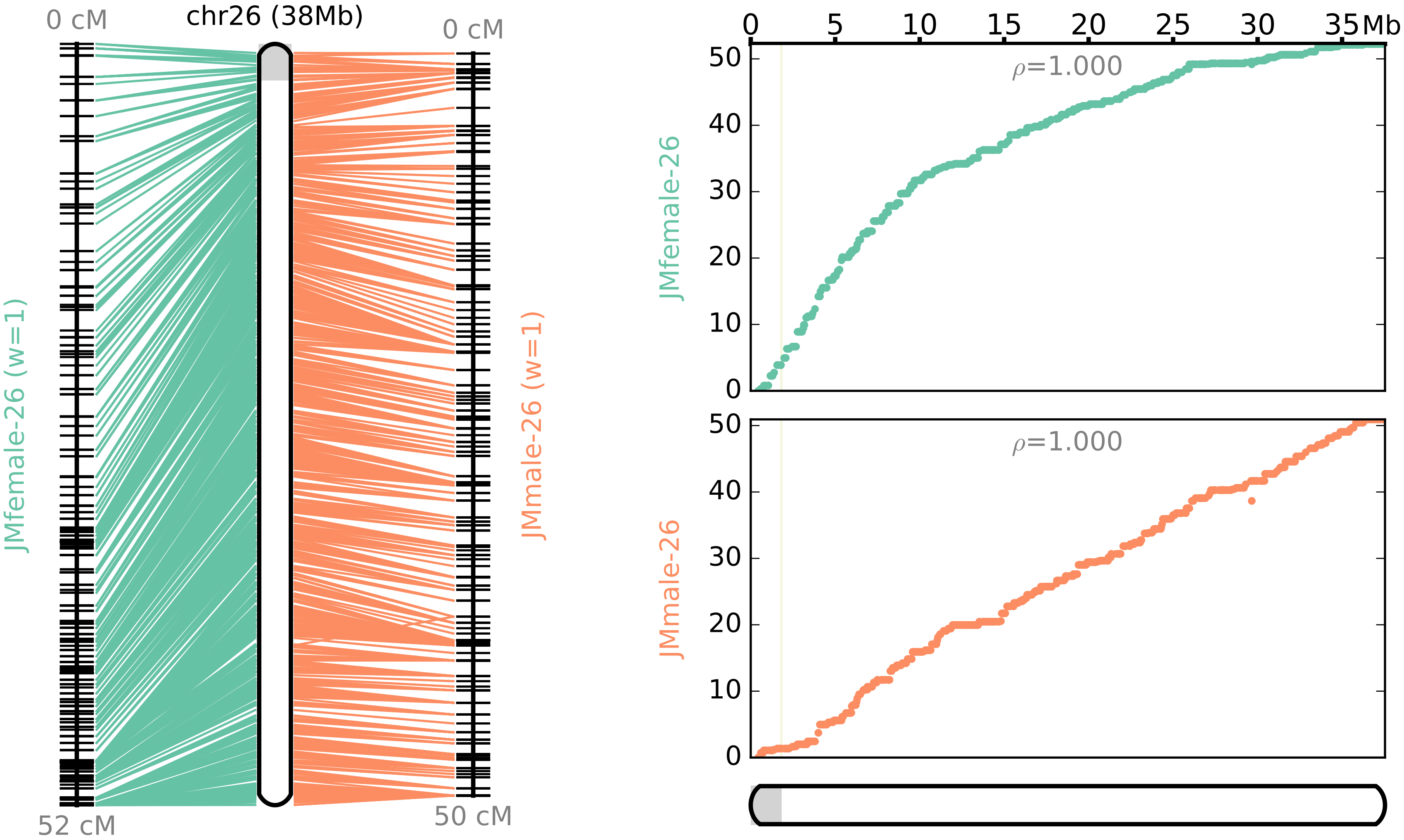
0 cM
chr26 (38Mb)
0 cM
JMfemale-26 (w=1)
JMmale-26 (w=1)
52 cM
50 cM
0
5
10
15
20
25
30
35Mb
50
40
30
20
10
0
ρ=1.000
JMfemale-26
JMmale-26

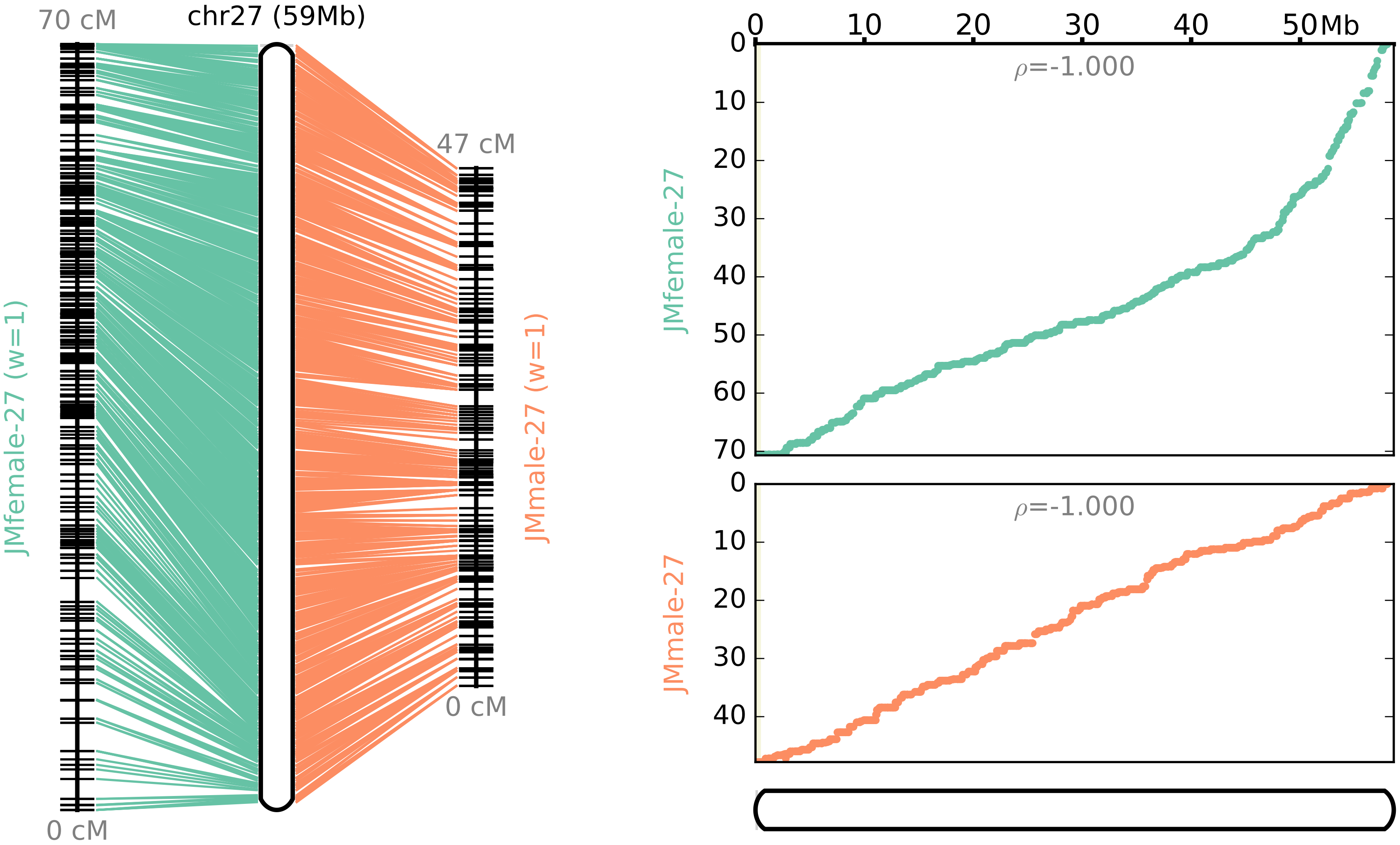

70 cM
chr27 (59Mb)
47 cM
0 cM
0 cM
JMfemale-27 (w=1)
JMmale-27 (w=1)
0
10
20
30
40
50Mb
ρ=-1.000
JMfemale-27
0
10
20
30
40
50
60
70
ρ=-1.000
JMmale-27
0
10
20
30
40

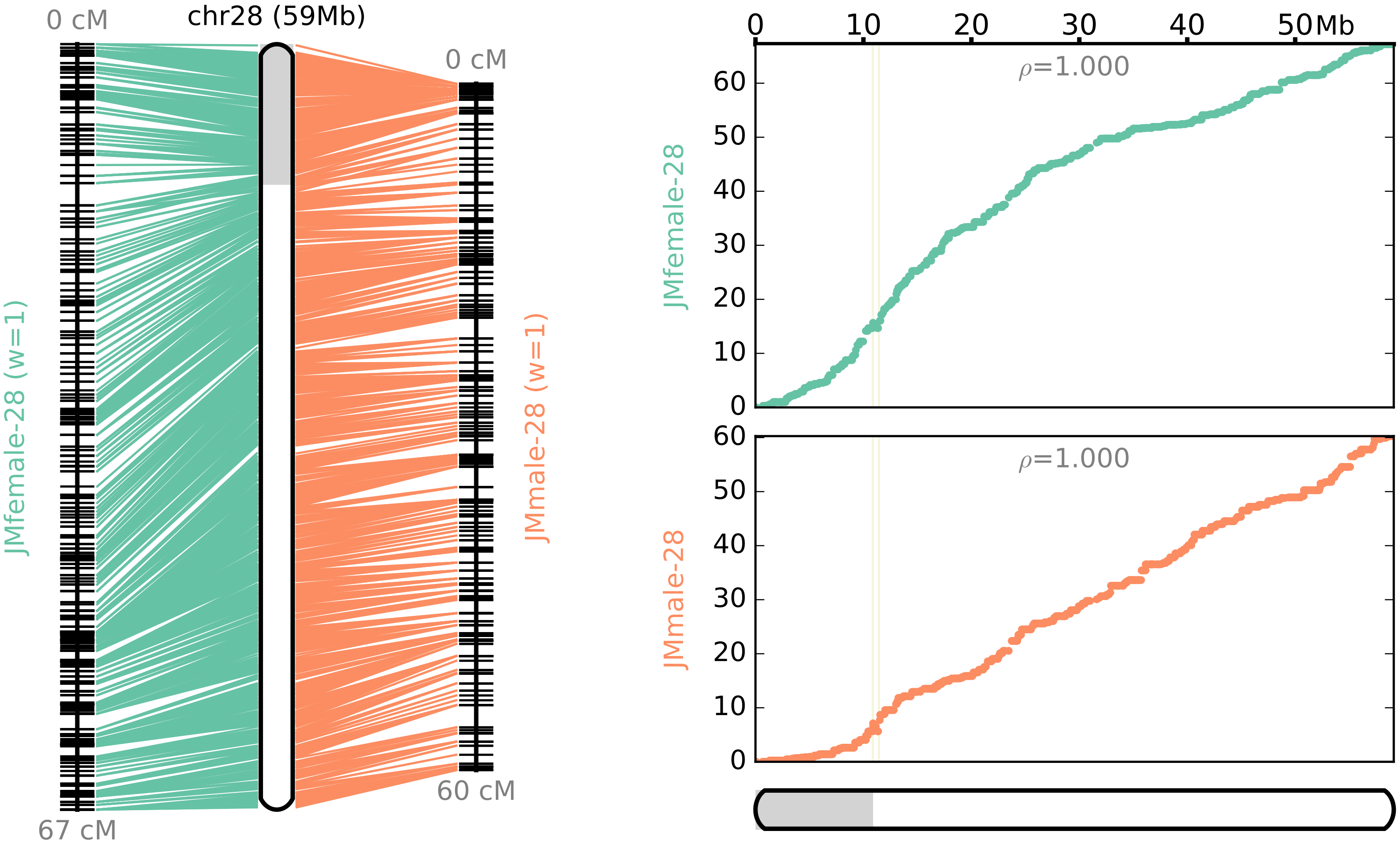

0 cM
chr28 (59Mb)
0 cM
JMfemale-28 (w=1)
JMmale-28 (w=1)
67 cM
60 cM
0
10
20
30
40
50Mb
ρ=1.000
JMfemale-28
0
10
20
30
40
50
60
ρ=1.000
JMmale-28
0
10
20
30
40
50
60

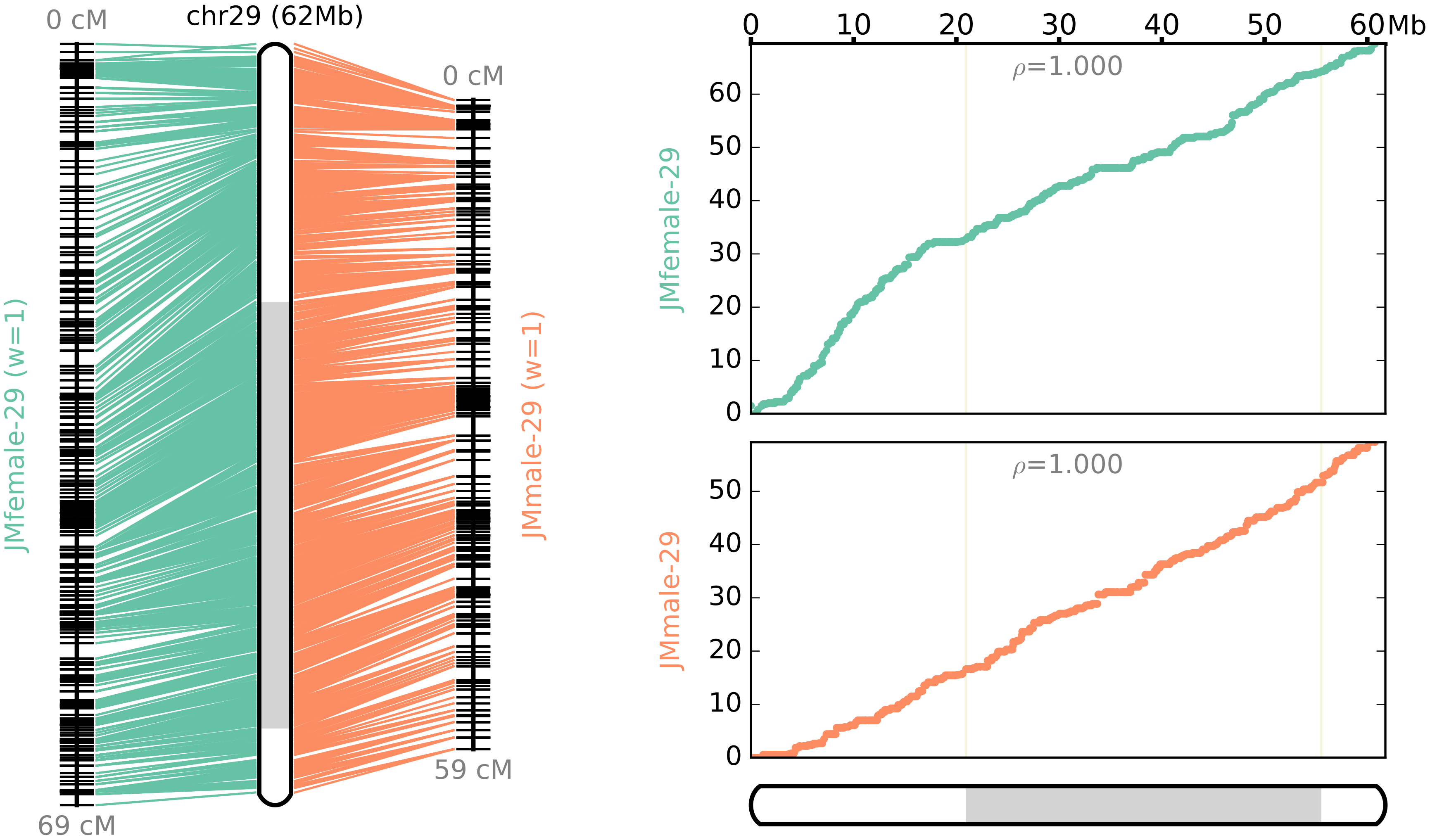

0 cM
chr29 (62Mb)
0 cM
69 cM
59 cM
JMfemale-29 (w=1)
JMmale-29 (w=1)
0
10
20
30
40
50
60
Mb
ρ=1.000
JMfemale-29
JMmale-29

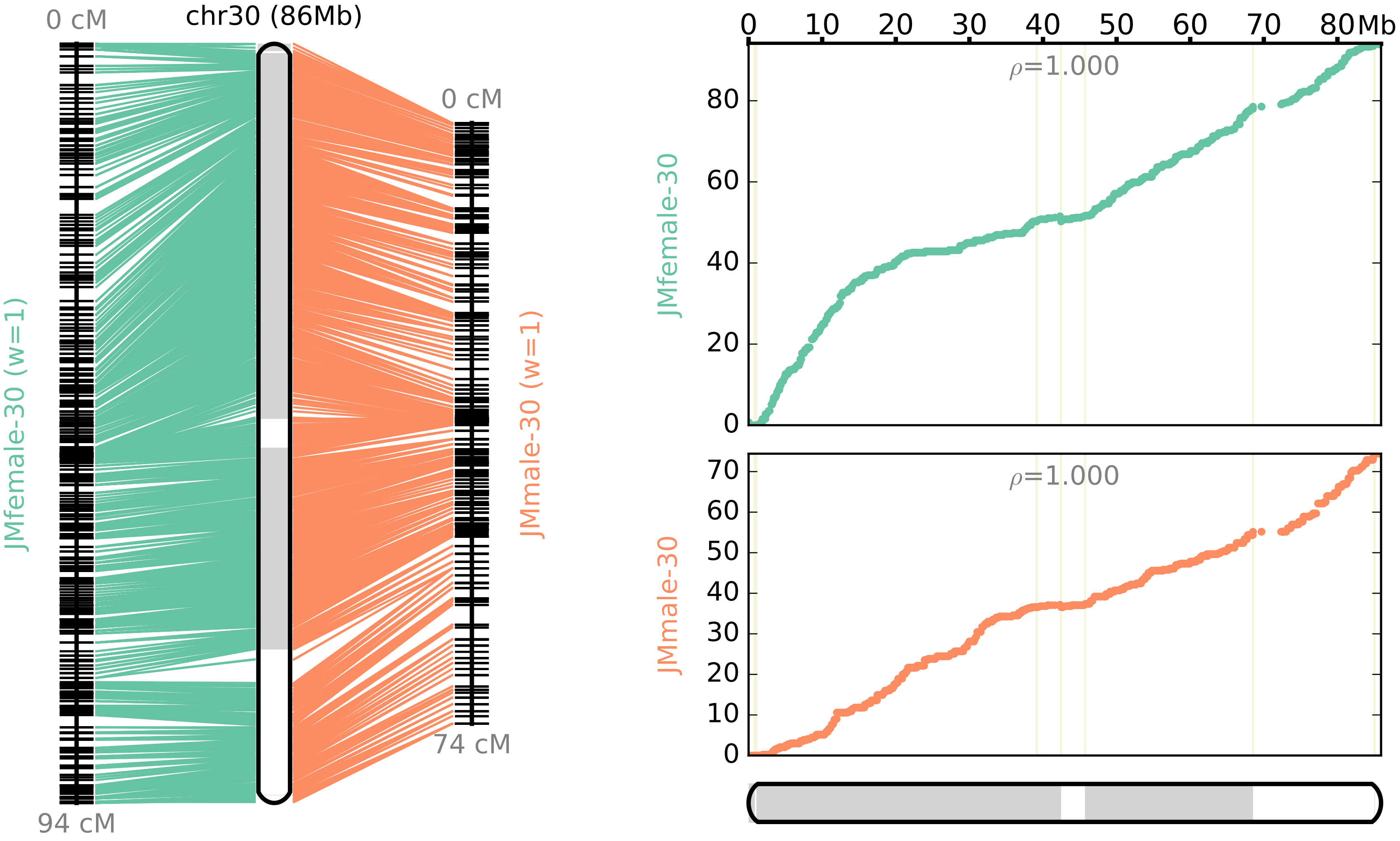

0 cM
chr30 (86Mb)
0 cM
74 cM
94 cM
JMfemale-30 (w=1)
JMmale-30 (w=1)
0 10 20 30 40 50 60 70 80Mb
ρ=1.000
JMfemale-30
0 20 40 60 80
ρ=1.000
JMmale-30
0 10 20 30 40 50 60 70

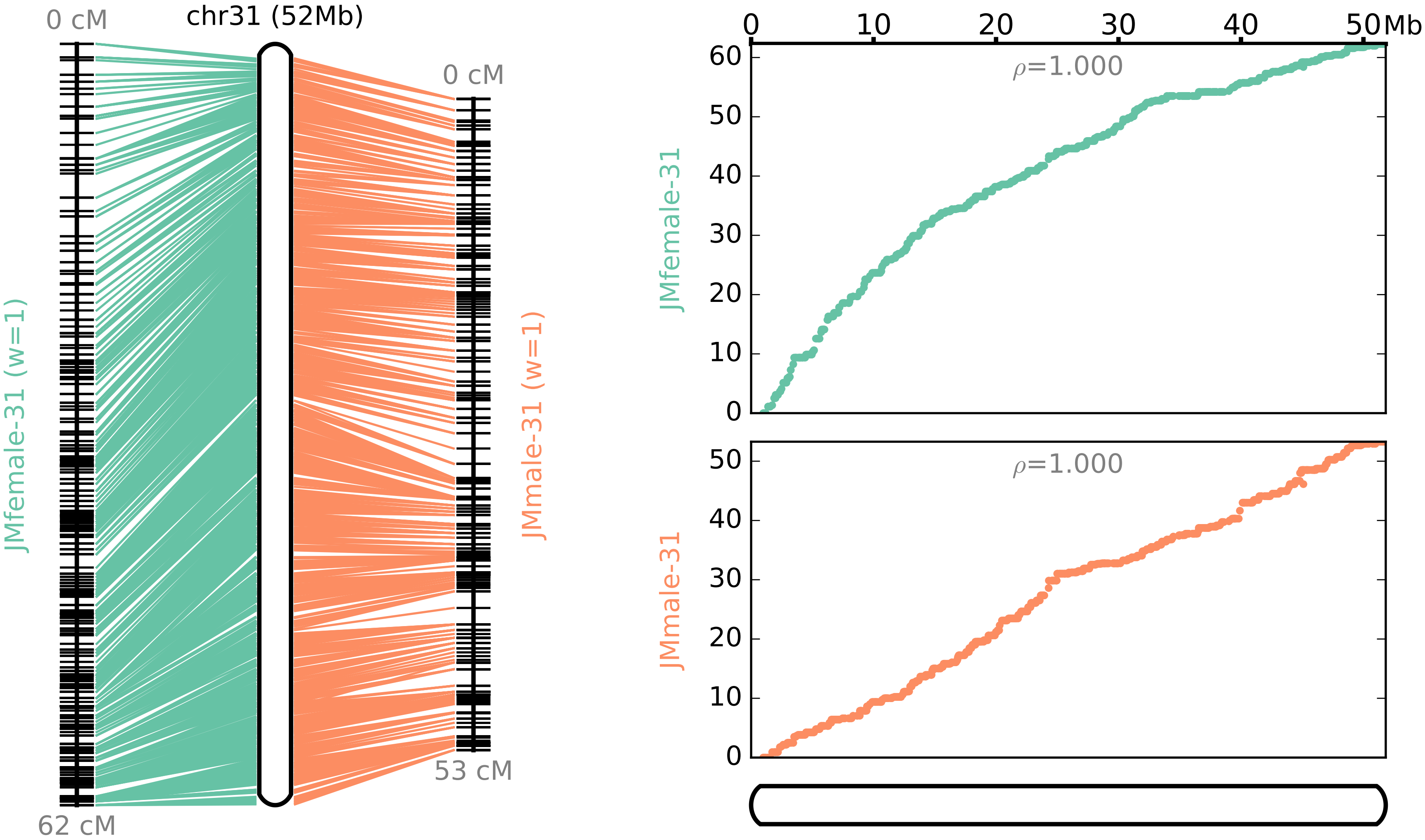

0 cM
chr31 (52Mb)
0 cM
JMfemale-31 (w=1)
JMmale-31 (w=1)
53 cM
62 cM
0
10
20
30
40
50Mb
JMfemale-31
ρ=1.000
60
50
40
30
20
10
0
JMmale-31
ρ=1.000
50
40
30
20
10
0

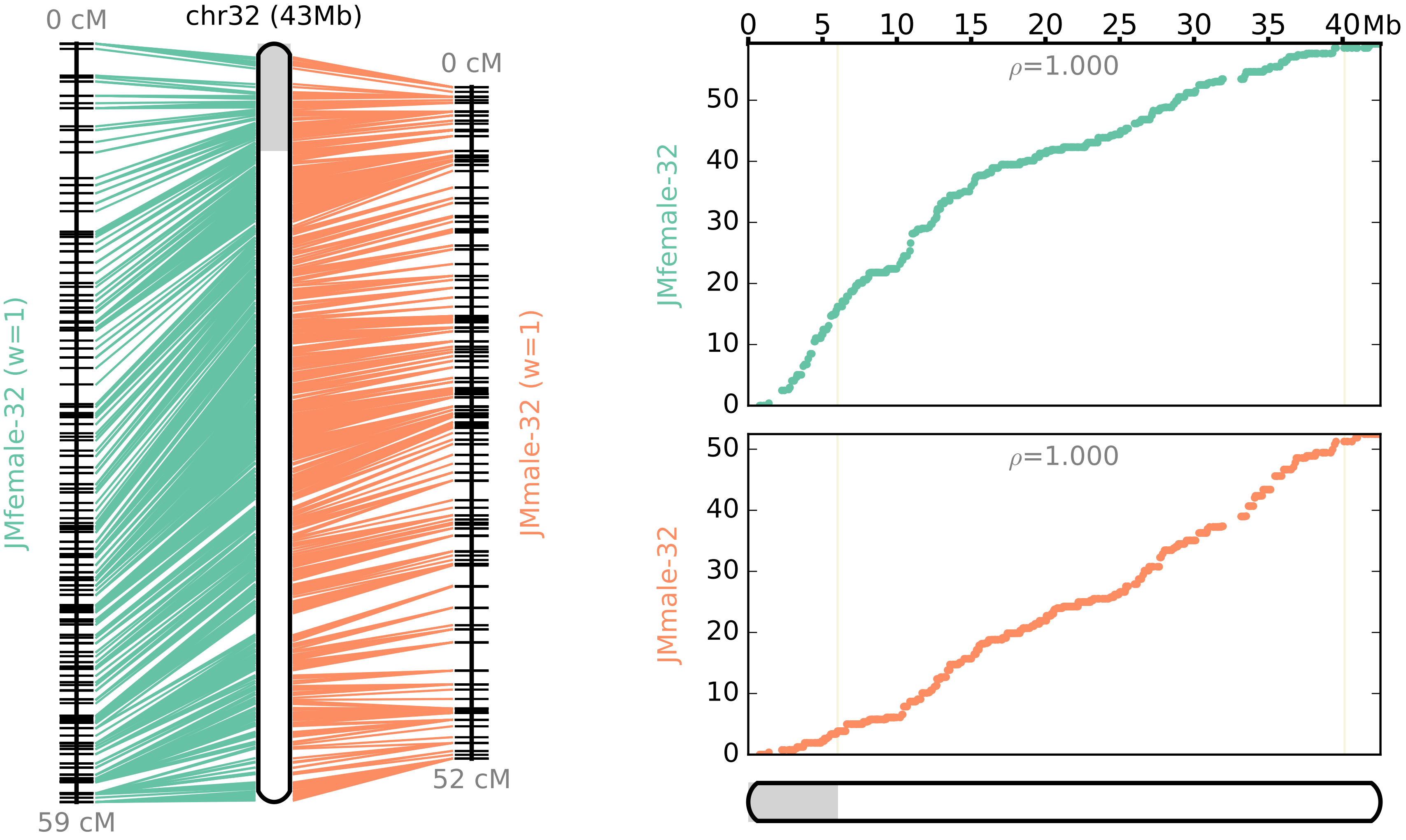

0 cM
chr32 (43Mb)
0 cM
JMfemale-32 (w=1)
JMmale-32 (w=1)
59 cM
52 cM
0 5 10 15 20 25 30 35 40 Mb
ρ=1.000
JMfemale-32
0 10 20 30 40 50
ρ=1.000
JMmale-32
0 10 20 30 40 50

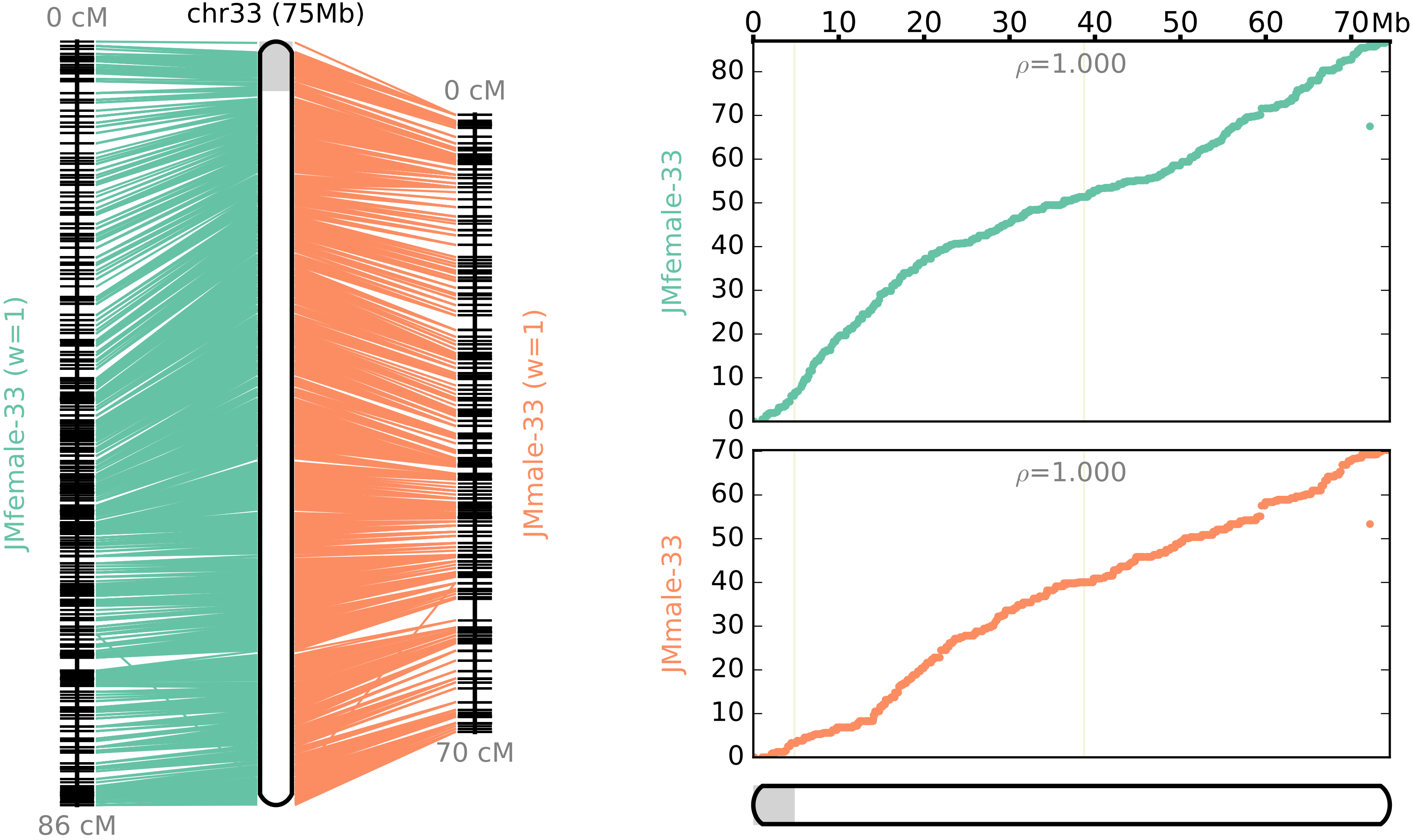

0 cM
chr33 (75Mb)
0 cM
JMfemale-33 (w=1)
JMmale-33 (w=1)
86 cM
70 cM
0
10
20
30
40
50
60
70Mb
ρ=1.000
JMfemale-33
80
70
60
50
40
30
20
10
0
ρ=1.000
JMmale-33
70
60
50
40
30
20
10
0

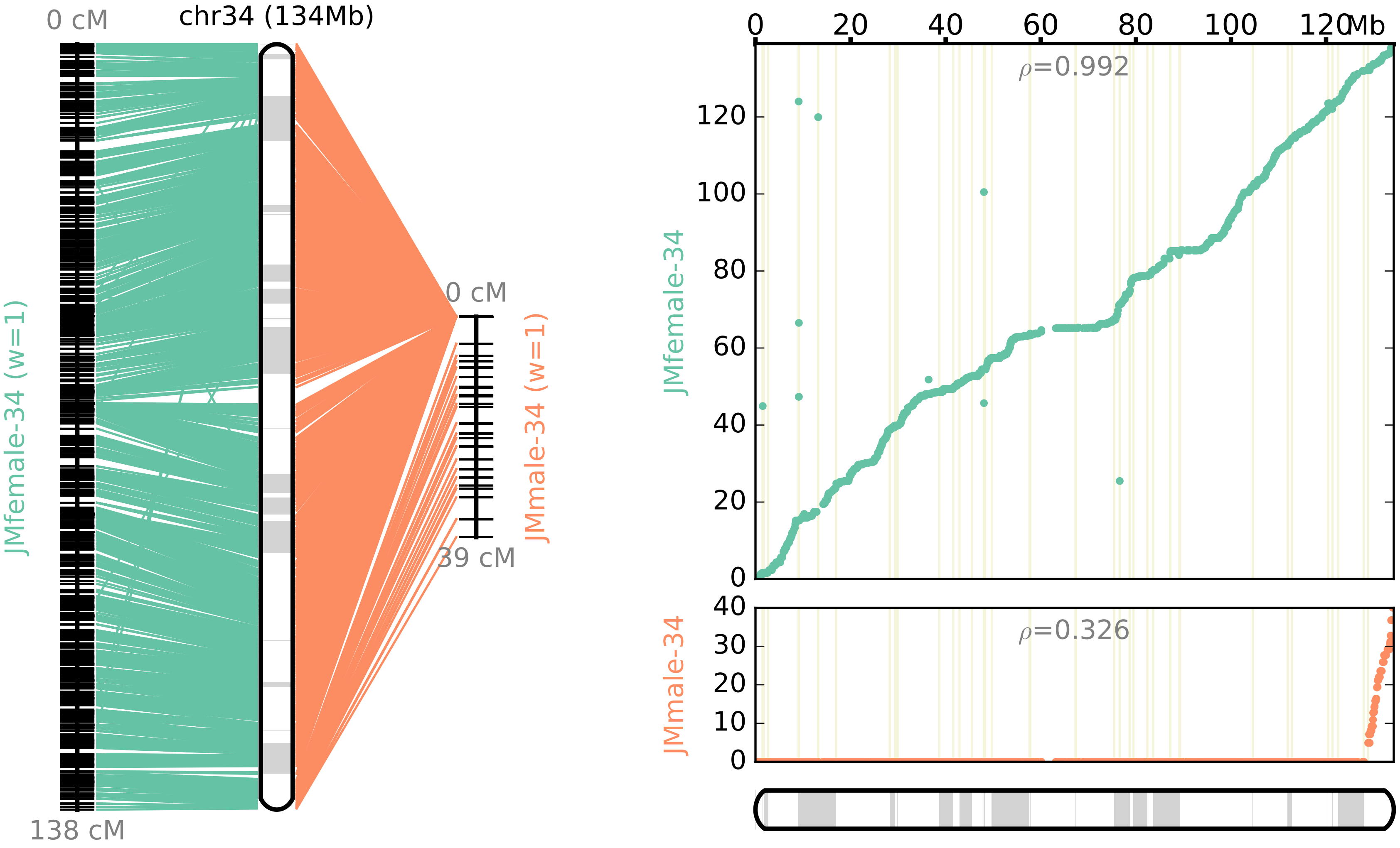

0 cM
chr34 (134Mb)
138 cM
JMfemale-34 (w=1)
0 cM
39 cM
JMmale-34 (w=1)
0
20
40
60
80
100
120
Mb
ρ=0.992
JMfemale-34
ρ=0.326
JMmale-34
